# Large Language Model–Based Agents for Physical Activity and Cognitive Training: Scoping Review

**DOI:** 10.2196/80123

**Published:** 2026-03-12

**Authors:** Alessandro Silacci, Benedetta Giachetti, Leonardo Angelini, Nicola Francesco Lopomo, Giuseppe Andreoni, Elena Mugellini, Mauro Cherubini, Maurizio Caon

**Affiliations:** 1 Department of Information Systems, Faculty of Business and Economics, University of Lausanne, Quartier Centre, Lausanne, 1015, Switzerland, 41 21 692 11 11; 2 Digital Business Center, School of Management Fribourg, HES-SO University of Applied Sciences and Arts Western Switzerland, Fribourg, Switzerland; 3 HumanTech Institute, School of Engineering and Architecture Fribourg, HES-SO University of Applied Sciences and Arts Western Switzerland, Fribourg, Switzerland, Fribourg, Switzerland; 4 Department of Informatics, Faculty of Science and Medicine, University of Fribourg, Fribourg, Switzerland; 5 Design Department, Politecnico di Milano, Milan, Italy; 6 Bioengineering Laboratory, Scientific Institute IRCCS E. Medea, Bosisio Parini, Italy

**Keywords:** cognitive training, conversational agents, large language models, physical activity, prompt engineering, reproducibility, scoping review

## Abstract

**Background:**

Large language model (LLM)–based conversational agents have been increasingly used in digital health interventions. However, their specific application to physical activity (PA) and cognitive training—two critical well-being domains—has not been systematically mapped. In fact, these domains share an important need for personalized, adaptive support and conversational engagement, making them relevant targets for examining how LLM-based agents are currently conceptualized and deployed.

**Objective:**

This scoping review aimed to map the extent, characteristics, and design practices of LLM-based conversational agents supporting PA or cognitive training, specifically analyzing their application contexts, social roles, and technological features.

**Methods:**

Following PRISMA-ScR (Preferred Reporting Items for Systematic Reviews and Meta-Analyses extension for Scoping Reviews) guidelines, we searched Web of Science, Scopus, PubMed, ACM Digital Library, and IEEE Xplore for studies published between January 2018 and December 2024. We included eligible studies that described LLM-based conversational agents designed for PA or cognitive training. Two reviewers independently screened records and extracted data. Descriptive synthesis and framework analysis were used to characterize intervention domains, agent roles, prompting strategies, model types, and reported outcomes.

**Results:**

Of 357 records screened, 10 studies met eligibility criteria (7 on PA and 3 on cognitive training). Applications predominantly involved coaching roles for PA and companion or scaffolding roles in cognitive domains. The agent landscape was dominated by proprietary LLMs (GPT-3.5, GPT-4, and Bard), with limited use of open-weight models. Prompt engineering emerged as a central yet inconsistently documented design mechanism. Reported outcomes mainly focused on perceived usefulness, engagement, or content quality, with few quantitative behavioral outcomes.

**Conclusions:**

LLM-based conversational agents have demonstrated early promise for supporting PA and emerging approaches to cognitive training, yet the current evidence remains exploratory and methodologically limited. Key challenges persist, including inconsistent reporting of prompts, reliance on proprietary models with limited reproducibility, and a lack of standardized outcome measures. More rigorous and transparently documented evaluations of these tools are required to strengthen the evidence base and guide future development.

## Introduction

### Background

Conversational agents (CAs) have been increasingly integrated into digital health interventions, offering scalable and personalized support for health-related behavioral change [1]. Within intervention domains such as physical activity (PA) promotion and cognitive health, CAs have reported promising results in fostering user engagement, supporting self-regulation, and enhancing adherence. These agents often mimic human dialogue to educate [2], prompt reflection [3], or guide users toward their behavior goals [4]. However, earlier conversational systems—particularly those based on prescripted or narrowly scoped interaction models—often failed to support the flexible, conversational interaction users expected, instead requiring command-like and highly constrained input that many users experienced as frustrating or unnatural [5].

The recent emergence of large language models (LLMs), such as OpenAI’s ChatGPT, introduces a fundamental shift in how CAs can be developed and deployed [6]. In fact, LLMs offer open-ended dialogue capabilities, context-sensitive responses, and general-purpose reasoning [7-9]—qualities that may greatly enhance the reach and efficacy of behavioral interventions. Early research suggests that LLM-powered agents can emulate counseling techniques, adapt their tone and content dynamically, and even facilitate therapeutic alliance-like interactions [10]. These affordances align with long-standing human-computer interaction (HCI) priorities, particularly the call for emotionally intelligent, adaptive, and personalized systems to support human well-being [11]. As such, LLM-based CAs are especially relevant in health domains where motivation, personalization, and sustained engagement are critical.

Despite the promise of LLMs, there is currently a lack of systematic understanding of how LLM-based CAs are being used, or could be used, in the context of PA and cognitive interventions. Existing literature reviews have examined artificial intelligence (AI)–powered CAs across domains, such as PA [12,13], obesity treatment [14-17], and mental health [18]. These studies highlighted several recurring limitations, including conversational rigidity, shallow personalization, limited contextual awareness, and repetitive or unnatural dialogue patterns [1,19]. Such shortcomings can hinder user engagement and learning effectiveness, particularly in interventions that rely on sustained motivation and adaptive feedback, as is the case for both PA coaching and cognitive training systems [20-22]. These weaknesses highlight a critical design gap that LLMs are poised to address, thanks to their capacity for open-ended interaction, adaptive tone, and flexible role construction. However, current reviews do not account for the distinctive capabilities, challenges, and design considerations introduced by this new class of models. As LLMs continue to gain traction in both academic and commercial health technologies, a timely synthesis is needed to chart the emerging landscape, identify key design patterns, and highlight open research questions.

Despite being distinct domains, PA and cognitive training are frequently integrated and combined in the literature, particularly within interactive and digitally mediated interventions [23]. Indeed, PA produces well-established cognitive and neurobiological effects [24]. Moreover, combined physical-cognitive training has been shown to yield synergistic benefits compared to single-domain approaches, although it remains subject to common challenges related to engagement, adherence, and intervention design. Recent interactive and exergame-based systems further demonstrate that physical and cognitive components are deeply intertwined in user interaction and system design, rather than implemented as isolated modalities [25]. Accordingly, this review considers both domains to examine how LLM-based CAs are designed and evaluated in well-being interventions. The focus is on their shared interactional, motivational, and personalization mechanisms, rather than on comparing clinical outcomes across domains.

This review addresses that gap by systematically mapping how LLM-based CAs are conceptualized, applied, and evaluated in interventions addressing PA and cognitive training. Our contribution is 2-fold. First, we characterize the state of the art in this fast-moving field, including system features, use contexts, and intended outcomes. Second, and more critically, we move beyond summary to dissect *how* these systems are built and the scientific challenges this creates. We specifically analyze the practice of prompt engineering as an informal yet central design mechanism. Furthermore, we highlight how the prevalent use of proprietary “black box” models and inconsistent documentation pose a fundamental threat to reproducibility, hindering the field’s cumulative scientific progress. By surfacing these methodological risks, we provide a necessary critical perspective that complements and advances prior work on digital health agents.

### Objectives

This scoping review explores the role of LLM-based CAs in supporting individuals’ PA and cognitive training, providing a comprehensive overview and critical evaluation of their impact. Specifically, it examines how these AI-driven agents facilitate engagement, personalize interactions, and address challenges in interventions aimed at enhancing both physical and cognitive well-being.

To structure this analysis, we investigated 3 key research questions (RQs), focusing on their applications, social dynamics, and integration with complementary technologies:

- RQ1. In what ways have LLM-based CAs been applied to support well-being, particularly in the contexts of PA and cognitive training?
- RQ2. How does existing literature characterize the social roles of LLM-based CAs in PA and cognitive training interventions?
- RQ3. What additional technologies or design features are integrated with LLM-based CAs to enhance their effectiveness in PA and cognitive training interventions?

## Methods

### Overview

This scoping review analyzes the landscape of LLM-based CAs for PA and cognitive training, following the PRISMA-ScR (Preferred Reporting Items for Systematic Reviews and Meta-Analyses extension for Scoping Reviews) guidelines (Checklist 1) [26] to enhance the transparency and completeness of reporting.

### Search Strategy

To identify relevant studies, keywords related to CAs, PA, cognition, and well-being were derived from a preliminary literature overview [27]. In parallel, LLM-specific keywords were selected based on prior LLM reviews and the authors’ domain expertise. Searches were primarily performed using combinations of these keyword groups (K1, K2, K3, ... Kn) across Clarivate Web of Science (WoS) and Elsevier Scopus, 2 databases recognized for their comprehensive coverage of peer-reviewed academic research [28,29]. To ensure comprehensive coverage, we further included PubMed, ACM Digital Library, and IEEE Xplore in our search based on their frequent use in prior literature reviews [27].

### Keywords and Queries

Keywords were collected based on other literature reviews’ requests involving agents used in PA and cognitive training. Queries included papers from January 2018 to December 2024, as this marks the introduction of Bidirectional Encoder Representations from Transformers, the first language model to enable bidirectional language understanding, a foundational feature for LLMs [30].

For full transparency and reproducibility, the complete and exact search query strings used for each database are openly accessible in a findable, accessible, interoperable, reusable (FAIR)–compliant repository [27]. As an example of our search structure, we used two main queries: one combining keywords from group K1 (agents), group K2 (technology), and group K3 (physical activity); and a second combining keywords from group K1 (agents), group K2 (technology), and group K4 (cognition), all using Boolean operators. A simplified representation of the search logic for these two distinct queries was as follows:

- Query 1: (*K1 keywords) AND (K2 keywords) AND (K3 keywords*)
- Query 2: (*K1 keywords) AND (K2 keywords) AND (K4 keywords*)

This structure was adapted to the specific syntax requirements of WoS and Elsevier Scopus.

### Study Selection

Inclusion criteria were established and adapted from previous research on CAs for well-being [31]. Results from query 1 and query 2 were uploaded to the Covidence [32] platform, where all authors were invited to participate in the review process.

The first selection phase assessed studies based on their title, abstract, and keywords, applying all inclusion and exclusion criteria except full-text availability and, in some instances, language. Following an initial pilot screening round (14 papers of which 5 were selected and 9 were irrelevant, ~36% eligible, details available as supplementary materials [27]), the inclusion and exclusion criteria were refined to improve clarity and consistency among reviewers. During the full-text screening phase, the complete set of eligibility criteria was applied (cf, Supplementary Material [27]). In both phases, each study was independently evaluated by at least two reviewers. In cases of disagreement, a third reviewer was consulted, and discussions were held when necessary to reach consensus.

### Data Extraction

Key study characteristics were extracted, including bibliographic details (title, authors, year, outlet or conference), study type, and specific information outlined in the data collection protocol available through our FAIR repository [27]. These included the study aim, LLM model and access modality, fine-tuning approach, characteristics of the CAs (name, form, role, purpose, and design), deployment context, interactional structure, software used, type of physical or cognitive activity, and prompt features. All data were independently reviewed by 2 authors. Discrepancies were resolved through discussion to ensure consistency and accuracy.

### Data Synthesis

To synthesize the collected data, we used both descriptive and qualitative approaches. Descriptive statistics were used to summarize key study characteristics, including publication year, country of study, study type and design, interaction modalities, and targeted domains (eg, PA and cognitive training). These metrics provided insights into the distribution and focus areas of existing research involving LLM-based CAs in the context of PA and cognitive training.

We used a framework analysis to synthesize findings from the included studies [33]. Two authors systematically extracted and charted textual data related to the design, use, and evaluation of the interventions. This charting focused on key domains, including the specific prompts used to guide the CAs and the types of outcomes measured (both qualitative and quantitative). This systematic approach allowed us to identify recurring patterns and emerging themes within the evidence base.

All conflicts or uncertainties in data interpretation were resolved collaboratively between at least two reviewers.

### Ethical Considerations

To ensure the utmost transparency and facilitate reproducibility, key research artifacts, specifically the full database search queries, the raw and consolidated data extraction datasets, and the R Markdown notebook used for quantitative analysis, as well as our framework analysis results have been publicly archived, adhering to the FAIR principles and recommendations provided by Niksirat et al [34]. The same supplementary material content is also available in the Multimedia Appendix 1.

## Results

### Search and Screening Results

**Figure 1.**
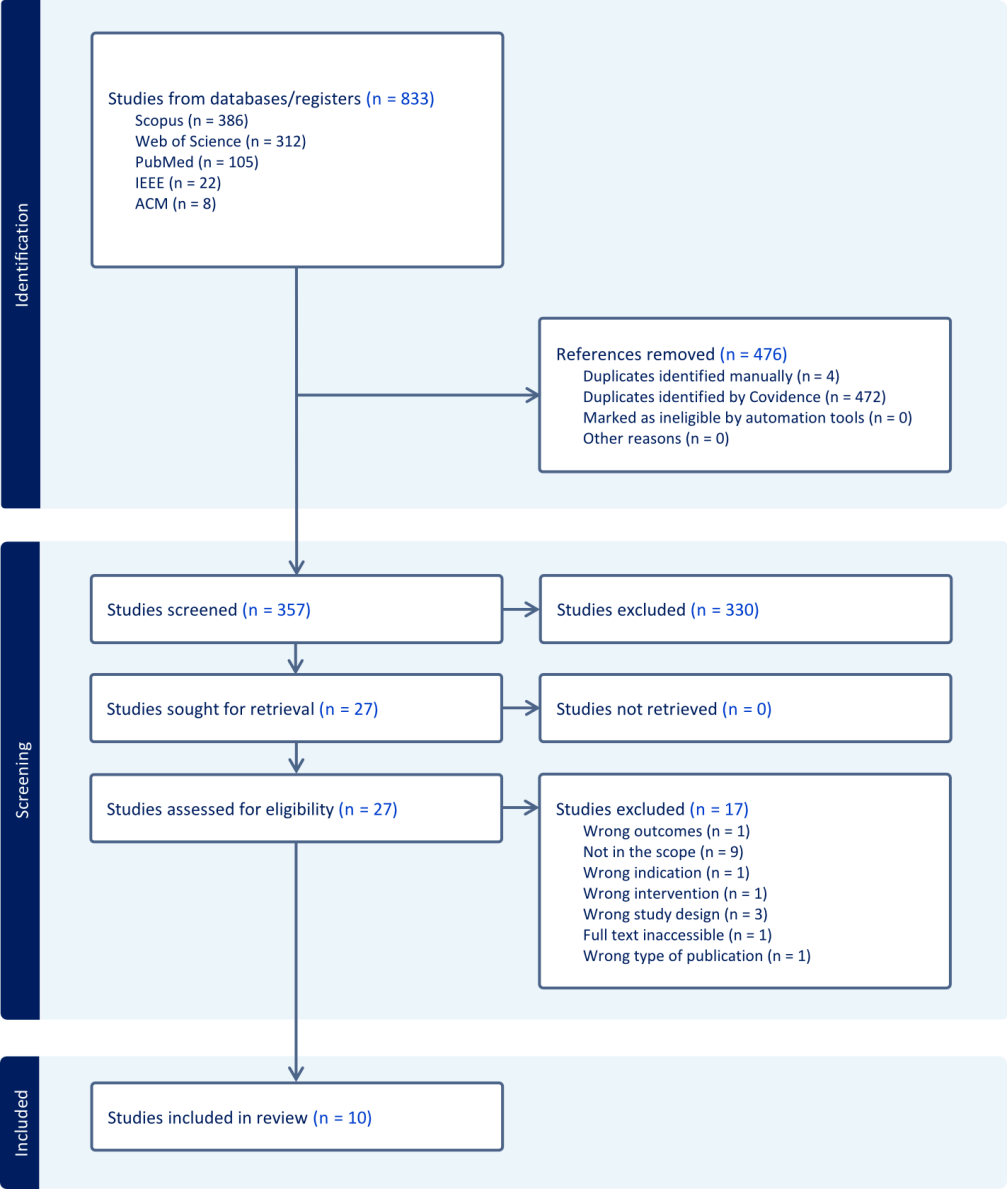
The PRISMA (Preferred Reporting Items for Systematic reviews and Meta-Analyses) flowchart, provided by Covidence, with the search and the selection process of the studies included in our review. ACM: Association for Computing Machinery; IEEE: Institute of Electrical and Electronics Engineers.

The initial database search yielded 833 records, with 386 (46.34%) from Scopus, 312 (37.45%) from Web of Science, 105 (12.6%) from PubMed, 22 (2.64%) from IEEE Xplore, and 8 (0.96%) from ACM DL. After removing 476 (57.14%) duplicates using Covidence, 357 (42.86%) records remained for title and abstract screening. Of these, 330 (92.44%) were excluded based on the inclusion criteria. The remaining 27 (7.56%) full-text articles were assessed for eligibility, resulting in the exclusion of 17 (62.96%) studies due to reasons such as being out of scope (n=9, 52.94%), wrong study design (n=3, 17.64%), or irrelevant outcomes, interventions, or indications. Ultimately, 10 (37.04%) studies met all eligibility criteria and were included in the final review (Figure 1). The dataset resulting from our analysis is available as supplementary material through our FAIR repository [27].

### Domains of Use and Contextual Focus

The 10 included studies explored a range of well-being applications, with a strong emphasis on PA (n=7; [35-41]), and fewer focusing on cognitive training (n=3; [42-44]). These interventions aimed to support behaviors such as exercise adherence, task planning, reminiscence, and memory stimulation. Most were delivered via web platforms (n=7; [35-39,42,43]), followed by mobile apps (n=2; [40,43]), messaging platforms (n=1; [41]), or robotic embodiments (n=1; [44]).

Several studies designed agents that adapted their responses to user input, preferences, or contexts— such as tailoring memory prompts, adjusting training plans, or offering reminders. In cognitive domains, agents facilitated engagement by drawing on familiar content (eg, book discussions, life stories), whereas in PA, personalization was often linked to goal tracking or motivation cues.

Most studies followed an empirical (n=7; [35,37-39,41-43]) or design-oriented (n=3; [36,40,44]) methodology, reflecting a pragmatic emphasis on system development and usability testing over theoretical modeling. However, only a few studies offered robust outcome measures.

These findings comprehensively address RQ1 by illustrating the primary domains (PA and cognitive training) and specific intervention goals (eg, exercise adherence, memory stimulation) where LLM-based CAs are currently being applied.

### Social Roles and Interaction Patterns

LLM-based CAs were designed with varied social roles, which ranged in both functionality and relational framing. The most common role was that of a personal coach [35-37,41], emphasizing directive and motivational engagement. Other roles included companions [43,44], assistants [40,44], counselors [38], experts [42], and recommender systems [39] (Table 1).

**Table 1.** The distribution of the roles that were attributed to the large language model–based conversational agents in the reviewed studies, demonstrating that these conversational agents were predominantly being used as coaches [35-37,41] but also involved as companions [43,44] and other more specific roles such as assistants [40,44], counselor [38], expert [42], and recommender systems [39].

Role	Reviewed articles
Personal coach	[35-37,41]
Companion	[43,44]
Scaffolding expert	[42]
Recommender system	[39]
Patient counselor	[38]
Digital assistant	[40,44]
Medical assistant	[44]

Despite this diversity, dialogue patterns remained limited: most systems were user initiated (n=5; [35,36,41-43]), with only [44] and [40] supporting shared initiative. Communication was primarily text-based [35-38,42,43], although 4 studies implemented voice interaction [40,42-44].

Another consistent pattern across all studies was the use of dyadic interaction models; indeed, the CA was always designed to interact with a single user at a time. No studies explored group dynamics, multiuser interfaces, or collaborative scenarios involving multiple stakeholders. This highlights a current boundary in the design space, where LLM-based CAs are predominantly framed as personalized, one-to-one agents rather than social actors embedded in broader systems or communities.

### Technological Design and Functional Integration

Technological integration centered around popular LLMs (Table 2), such as GPT-4.0 (n=5; [36,37,40,43]) and GPT-3.5 (n=4; [35,36,39,42]), with occasional use of alternatives such as Google Bard [35,40], Mistral [35], and Llama [35,43]. These models were most commonly accessed via web-based interfaces, with several studies directly using web clients such as ChatGPT’s web platform [45], and some others integrated the models into mobile applications. A smaller subset of studies incorporated multimodal features, such as voice interaction, embodied agents (eg, the EVA robot [46]), or context-aware elements such as memory recall cues, directly detailing the additional technologies and design features specified in RQ3.

**Table 2.** A reporting of the different large language models used in the reviewed studies with a main use of GPT-4.0 (n=5; [36,37,40,41,43]), GPT-3.5 (n=4; [35,36,38,39]), and Google Bard [35,40] for the closed-source models, in addition to the mention of open-weight models, such as Mistral [43] and Llama [35,43].

Developer, model, and variant	Reviewed articles
OpenAI	
ChatGPT	
GPT-3.5	[35,36,38,39]
GPT-3.5-turbo	[42]
GPT-4.0	[36,37,40,41,43]
NA	[44]
Whisper	[44]
Google	
Bard	
NA	[35,40]
Meta	
Llama	
2	[35]
2-13B	[43]
Mistral.ai	
Mistral	
7B	[43]

A cross-cutting category was related to the use of engagement and motivation strategies, including adaptive scaffolding, goal reminders, or user-personalized prompts. While some studies integrated these intentionally, others relied on the natural language capabilities of LLMs to simulate engagement (eg, responding conversationally or using humor). Only a subset of studies reported evaluating these engagement-related elements in a structured way, while others did not include specific assessments of these components.

Finally, many studies highlighted technical or operational challenges. These included LLM response delays, platform limitations, and concerns over content appropriateness or factuality. Several studies used semiautomated systems due to constraints in LLM access or stability. Moreover, the lack of standardized outcome measures was a consistent limitation: although most studies reported user satisfaction or feasibility, few provided behavioral or cognitive metrics tied to intervention efficacy.

### Prompting as Design: Framing Roles, Personalization, and Interaction

In addition to model selection and platform deployment, the reviewed studies revealed a critical design layer in the form of prompt engineering—that is, the crafting of textual inputs that guide the behavior, tone, and identity of LLM-based CAs. Although rarely emphasized as a formal methodology, prompt design emerged as a powerful mechanism through which researchers shaped the agent’s functional logic, social role, and interpersonal dynamics. The resulting framework analysis of the prompt strategies observed across the included studies highlights 4 key patterns in how prompts were used to construct agent behavior and interaction style (Table 3).

**Table 3.** A summary of the different categories that emerged when analyzing the provided prompts in the reviewed studies (7 papers provided prompts out of the 10 analyzed).

Category	Studies
Instructional and informational requests	[36,37,39,42,44]
Role-based identity assignment	[42-44]
Scenario-based personalization	[36,42-44]
Task-oriented dialogue support	[42,44]

Across the studies, prompts were not generic queries but scripted scenarios or instructions that encoded interactional intent. In many cases, this took the form of scenario-based personalization. For instance, Favela et al [44] framed interactions within dementia care routines, embedding the agent in emotionally significant and context-sensitive dialogue.

Prompting also played a decisive role in constructing the social identity of the agent. Multiple studies used explicit role-based instructions, instructing the LLM: “You are a book club host,” “You are a healthcare assistant,” or “You are a caregiver.” These role assignments acted as social anchors, shaping how the CA would behave—more directive as a coach [36], more empathetic as a companion [44], or more informative as a recommender system [39]. These framing devices reflect a broader reliance on prompting to simulate relational presence, particularly in the absence of embodied or affective sensing.

In terms of functionality, prompts often scaffolded task-oriented dialogues. For example, Hu et al [42] embedded conditional logic into its prompt to simulate a grocery shopping task for cognitive assessment. Rather than scripting full interaction flows, these designs leveraged the LLM’s interpretive flexibility, using natural language to create interactive, multistep tasks without formal programming and prompting thus served as a low-code interface for designing agent behavior.

Some prompts explicitly addressed affective tone and ethical conduct. Favela et al [44] instructed the CA not to “talk like a child” when interacting with older adults and to remain “patient and respectful.” Such affect-aware framing suggests a growing awareness that prompts are not only functional but also relational instruments, capable of shaping the user’s emotional experience.

Importantly, all studies used LLMs in dyadic contexts, with prompts structured for one-on-one interaction. No study designed prompts to support group conversation, multiuser turn-taking, or collective memory tasks. This reflects a current boundary in the field: despite LLMs’ flexible dialogue capabilities, they are still being operationalized primarily as personalized single-user agents.

Of the 10 reviewed studies, 3 did not provide the prompts in the text or the supplementary materials. One additional study [35] did not use prompting to shape the agent’s behavior as a CA but instead framed the task as a reasoning exercise: the LLM was given a user profile and asked to infer likely future decisions from that perspective.

Taken together, the analysis of prompt strategies highlights that prompting is not just a technical necessity but a central design practice. Whether used to construct social roles, personalize content, or manage conversation flow, prompts serve as the invisible scaffolding behind agent behavior. Yet, few studies evaluated or iterated on prompt effectiveness, suggesting a need for more systematic approaches to prompt design and testing in future work.

The analysis of prompt strategies highlights that prompting is not just a technical necessity but a central design practice. Whether used to construct social roles (RQ2), personalize content, or manage conversation flow (both enhancing effectiveness, RQ3), prompts serve as the invisible scaffolding behind agent behavior.

### Reported Outcomes: Perception, Behaviors, and Evaluation Gaps

The included studies reported a range of qualitative and quantitative outcomes, offering insights into both user experience and the effectiveness of LLM-based CAs. A framework analysis of these outcomes revealed five dominant categories: perceived usefulness, user engagement, content quality, behavioral impact, and a notable lack of quantitative evaluation. These categories illustrate how CAs are currently being evaluated and highlight where the evidence remains limited or anecdotal (Table 4).

**Table 4.** A summary of the different categories that emerged from the outcomes’ analysis in the reviewed papers.

Category	Studies
Behavioral impact	[35,41]
Content quality	[37-39]
Perceived usefulness	[36,42]
User engagement	[41,42,44]
Lack of quantitative evaluation	[36,37,39,40,43]

Perceived usefulness was a recurring category in the reviewed papers. For instance, participants in the study by Hu et al [42] described the agent as helpful and easy to use, indicating a positive perception of its utility in their tasks.

Similarly, user engagement was frequently reported as a key outcome. Several studies used interaction metrics as a proxy for engagement; Favela et al [44] noted that users sustained conversations lasting for over 10 minutes, suggesting a naturalistic and engaging interaction. Hu et al [42] documented consistent use of specific features, such as recommendations, while Sun et al [41] found that exposure to the agent was a significant predictor of engagement. The latter study also identified specific design elements, such as humor, as potential enhancers of engagement, although their direct motivational impact was not conclusively determined.

The content quality of the agent’s output emerged as a dual-focused category, with studies reporting both strengths and significant weaknesses. On the positive side, Washif et al [36] found that an agent’s exercise recommendations were consistent with standard plans. Bak and Chin [35] reported that LLMs could generate more stage-appropriate health information when provided with user profiles that included clear goals. However, the same study noted limitations in the recommendations for users in certain stages of behavior change. Some studies reported several limitations related to the accuracy, reliability, or personalization of the agents’ responses. Pugliese et al [38], for example, found that while responses were understandable, not all information provided was reliable or sufficiently personalized.

A smaller subset of studies attempted to measure behavioral impact, although often with preliminary or indirect indicators. Bak and Chin [35] evaluated the potential for LLMs to address different stages of the transtheoretical model, highlighting that the models tended to favor certain strategies over others without providing clear reasons. While not finding significant changes in motivation, Sun et al [41] did demonstrate that the inclusion of humor had a measurable effect on participants’ PA, pointing to a potential, albeit subtle, behavioral influence.

Finally, a critical finding was the widespread lack of quantitative evaluation. While a few studies reported quantitative metrics such as conversation time [44] or feature usage [42], these were the exception. Many papers explicitly acknowledged that their evaluation was exploratory or provided no quantitative data at all [36,37,39,40,43].

Overall, the limited application of standardized outcome measures suggests that the current evidence base remains preliminary. While studies frequently reported positive user perceptions, few included validated behavioral or cognitive assessments to substantiate claims of effectiveness. This constrains the ability to draw firm conclusions regarding both the impact of these systems (RQ1) and the design choices that shape their performance (RQ3).

## Discussion

### Principal Findings

This scoping review examined how LLM-based CAs have been applied to support well-being, with a focus on PA and cognitive training. Through framework analysis, prompt categorization, and evaluation of reported outcomes, we identified patterns in how these systems are designed, deployed, and assessed. In the following sections, we interpret the findings across three key areas: application contexts, role construction, and design and evaluation strategies.

First, in terms of application contexts, our results show that LLM-based CAs are primarily deployed in interventions related to PA, where they function as digital coaches offering motivational prompts, personalized planning, and behavioral reinforcement [35,36]. These roles align with traditional coaching models that emphasize goal setting, encouragement, and self-monitoring [47]. In contrast, cognitive training applications remain limited and exploratory. The 2 studies in this domain focused on reminiscence [44] and task scaffolding [42] rather than delivering structured cognitive exercises grounded in validated protocols.

This pattern suggests that PA interventions may present a more immediate design fit for LLM-based agents, particularly because motivational dialogue can be framed using general-purpose language generation without requiring deep domain modeling, thereby furthering our understanding of RQ1. Conversely, cognitive interventions—especially those targeting impairments—require higher precision, domain knowledge, and ethical sensitivity, which current prompt-based implementations may struggle to provide. Moreover, most applications in both domains rely on static personalization, often configured during initial sessions, rather than dynamically adapting to user behavior or outcomes over time.

These findings highlight an underexplored opportunity to use LLM-based agents for more structured cognitive support. Future work should explore how these systems can be embedded in adaptive frameworks that respond to longitudinal user behavior, particularly in contexts such as memory training or executive function support. Additionally, the absence of evidence-based cognitive training protocols underscores the need for interdisciplinary collaboration between HCI, cognitive neuroscience, and clinical design.

Our review shows that LLM-based CAs predominantly rely on prompt-engineered instructions to instantiate social roles such as “coach,” “companion,” or “caregiver.” This design choice enables rapid prototyping; however, it also exposes a deeper structural limitation. Indeed, LLMs do not maintain stable personas across extended interactions [48,49]. Recent empirical work demonstrates that persona adherence degrades during multiturn dialogue, with models gradually drifting away from assigned psychological profiles or communicative styles. Bhandari et al [49] show that LLMs frequently lose alignment with Big Five trait configurations over the course of dyadic conversations, even when such traits are explicitly embedded at initialization, highlighting inconsistencies in sustained personality expression. Similar instability has been documented in emotional support settings, where personas influence strategy use but undergo measurable shifts in emotionality and extraversion as conversations unfold [50]. Once a user diverges from the scripted interaction, the role coherence can collapse, exposing the user to potential agent hallucinations [51]. This observation is consistent with findings in the LLM literature showing that persona conditioning degrades over time without architectural support (eg, memory, state tracking) [52].

This fragility aligns with theoretical perspectives on role-play in LLMs, which conceptualize dialogue agents as enacting simulacra that lack internal persistence. Shanahan et al [53] argue that LLMs “role-play” characters by following statistical patterns rather than maintaining grounded identities; consequently, persona continuity is inherently brittle in the absence of architectural mechanisms such as memory or state tracking. Our findings similarly reflect that current well-being–oriented CAs rarely incorporate such mechanisms, relying instead on static instructions that do not support evolving or contextually reinforced identities.

Furthermore, research in HCI and communication science indicates that personas—especially when used to guide social interaction—impact user perception, engagement, and trust. Controlled experiments with embodied LLM agents show that manipulating personality traits (eg, introversion vs extraversion) significantly affects social evaluations, emotional experience, and behavioral engagement [54]. However, these effects depend on the consistency and credibility of the persona. Complementary evidence reveals that AI-generated personas often appear stereotypical or insufficiently nuanced compared to human-crafted ones, raising concerns about whether LLM personas authentically capture user diversity or complexity [55]. Similarly, work on demographic persona prompting demonstrates that LLMs may reflect demographic biases or fail to accurately maintain demographic-specific viewpoints unless tightly constrained [48].

This lack of continuity is critical because it undermines the conditions under which users treat agents as social actors. Nass and Moon [56] demonstrated that humans apply social norms to computer agents when those agents maintain a coherent identity and consistent interpersonal behavior—qualities that foster deeper engagement and trust. These effects rely on interactional consistency and memory to simulate “mindfulness” and sustained social presence, both of which are often absent in current LLM-based implementations. This challenge was also highlighted by Pataranutaporn et al [57], who note that although AI-generated characters can simulate highly personalized roles (eg, mentor and therapist), their relational integrity is fragile unless backed by memory systems and contextual continuity mechanisms.

Taken together, these insights suggest that prompt-based social role construction is brittle and insufficient for sustained engagement. To address this, future systems should incorporate persistent memory mechanisms, state awareness, or hybrid logic layers to reinforce social cues across interactions. Moreover, there is a clear need for empirical evaluation of persona coherence—an area that remains largely untested despite its centrality to trust, compliance, and user satisfaction in relational agents.

The third major theme emerging from our findings concerns the profound challenge that current design practices pose to scientific rigor. LLM-based CAs reflect a tension between design flexibility and methodological fragmentation. On the one hand, the ability to craft prompts as low-code design primitives allows for rapid customization and iterative prototyping. Developers can create context-sensitive interactions by embedding goals, roles, and emotional cues directly into natural language instructions. However, as recent work has shown, this flexibility often comes at the cost of consistency, transparency, and evaluative rigor. Large-scale methodological reviews increasingly describe LLM research ecosystems as fragmented and underspecified, noting that the lack of shared standards for documenting prompts, configurations, and evaluation pipelines produces substantial barriers to comparability and scientific accumulation [58,59].

For instance, Hanauer et al [60] found that a large proportion of LLM-driven clinical studies failed to report basic implementation details, such as model version, parameter settings, or the timing of usage—critical factors that undermine reproducibility. Similarly, Zamfirescu-Pereira et al [61] demonstrate that non-AI experts often struggle to design effective prompts and rarely document prompt rationale, iterations, or failures. These studies highlight that while prompt engineering lowers technical barriers, it introduces new challenges related to reproducibility, replicability, and responsible design practice. Prompt design is rarely subjected to empirical testing, and its impact on system behavior is often undocumented or informally evaluated—a concern echoed in psychological research, where Demszky et al [62] warn that LLM-based interventions frequently lack theoretical grounding or validation against established constructs.

This lack of formalization extends directly to evaluation practices. Few studies attempted to link agent interaction with behavioral or cognitive outcomes, and fewer still used validated instruments. Overall, the variability and limited rigor of outcome measures across studies suggest that much of the current work remains at a proof-of-concept level, with an emphasis on feasibility and system development rather than validated behavioral or cognitive outcomes. These observations are consistent with findings from Shool et al [63], who reviewed more than 700 LLM studies in clinical medicine and found that most relied on ad hoc performance indicators—such as accuracy or readability—while neglecting more robust, psychometrically validated tools. These observations echo broader concerns in recent LLM evaluation scholarship regarding the limitations of current benchmarks and metrics, which insufficiently capture meaningful, real-world performance or safety [58].

These combined observations on design and evaluation raise important concerns about the current maturity of LLM-based CA research in well-being domains. To strengthen the evidentiary base, future work should adopt mixed methods designs with validated outcome measures and comparative baselines. Furthermore, prompt design artifacts, agent configurations, and transcripts should be published or shared where possible to support reproducibility and transparency in this rapidly evolving design space.

Finally, beyond the challenges of prompt transparency, a deeper impediment to reproducibility in LLM-based interventions lies in the widespread reliance on proprietary, continuously updated models. These systems—such as OpenAI’s GPT series and Google’s Gemini—are fundamentally nontransparent black boxes, a characteristic repeatedly highlighted in the literature as a central obstacle to accountability and scientific verification. Existing literature emphasizes that LLMs’ internal mechanisms remain opaque even to expert users, complicating efforts to understand or trace how outputs are generated (eg, their “algorithmic opacity”) and limiting the ability to contest or replicate results [64,65].

A critical consequence of this opacity is model drift. Indeed, proprietary LLMs are routinely updated without version-locking or archival access, meaning that the same prompt issued weeks or months apart may yield measurably different outputs. Such evolving behavior has been noted as incompatible with basic scientific principles of repeatability and falsifiability, as researchers cannot access, “freeze,” or independently inspect prior states of the model used in their studies [66]. This challenge is distinct from issues of explainability; even perfect prompt documentation cannot compensate for the fact that the underlying computational pathway is inaccessible and mutable.

The inability to audit or update underlying training data compounds this problem. While transparency frameworks increasingly stress the importance of auditability (ie, the ability to identify what data or processes contributed to an output), current proprietary LLMs rarely enable such inspection, creating structural barriers to verifying results or correcting errors [66]. Scholars further warn that focusing solely on explainability can obscure the more pressing practical issue: users lack sufficient clarity about how these systems operate and what their limitations are, further undermining reproducibility across contexts [67].

Given these constraints, researchers using proprietary LLMs should explicitly acknowledge the inherent limitations to reproducibility and document, at minimum, the date range of model access, application programming interface version information (if any), and all implementation details that can feasibly be reported. Although such documentation cannot fully compensate for the absence of model version stability, it substantially improves transparency and allows future researchers to contextualize observed outputs within the dynamic evolution of the model.

In addition to transparency constraints, reproducibility poses an even more fundamental challenge for LLM-based research. Recent evaluations across clinical and information-retrieval domains reveal that proprietary LLMs exhibit intrinsic output instability: even when prompts, inputs, and contexts are held constant, model responses vary in ways that cannot be fully controlled or accounted for by researchers [68,69]. This instability reflects not simply stochastic sampling, but deeper properties of opaque, continuously optimized systems whose internal states and inference pathways are inaccessible. As a result, reproducibility failures arise even before model drift is considered, compounding the challenge introduced by unannounced backend updates. Together, these characteristics make LLMs fundamentally different from traditional research instruments: they cannot be frozen, independently audited, or deterministically rerun. Consequently, LLM-based studies must treat reproducibility not as a procedural hurdle but as a structural limitation of the technology, necessitating explicit acknowledgment and meticulous reporting of model versions, access dates, prompt configurations, and variability observed during experimentation.

### Limitations

While this scoping review provides a comprehensive mapping of LLM-based CAs in PA and cognitive training, it is important to acknowledge several limitations that shape the interpretation and generalizability of our findings.

As a scoping review, our primary aim was to map the breadth of existing literature rather than to conduct a deep synthesis or formal quality appraisal of individual studies. Consequently, we did not formally assess the methodological quality or risk of bias of the included studies, meaning our review cannot make definitive statements about the robustness of the evidence or the causal effectiveness of the interventions. Similarly, we did not perform a meta-analysis or other quantitative aggregation of outcomes due to the inherent heterogeneity of study designs, interventions, and outcome measures, with our reported outcomes primarily consisting of qualitative summaries. While providing a comprehensive overview, the broad nature of a scoping review also implies that specific nuances within individual studies or particular application contexts might not have been explored in exhaustive detail.

Our search strategy, while comprehensive within its defined parameters, was subject to certain constraints. Searches were limited to WoS and Elsevier Scopus; thus, relevant studies published in other databases, gray literature, or conference proceedings not indexed in these sources might have been missed. The effectiveness of our search was also dependent on the chosen keyword combinations (K1, K2, K3, and K4); although these were developed through preliminary scans and expert consultation, it is possible that alternative terminology or emerging concepts related to LLM-based CAs, PA, or cognitive training were not fully captured. Furthermore, our review was restricted to articles published in English, potentially excluding pertinent research published in other languages. While justified by the emergence of LLMs, the time frame restriction to publications between January 2018 and December 2024 means that earlier foundational work or very recent developments (post-2024) were not included. Finally, despite a comprehensive search, the small sample size of only 10 studies meeting our stringent eligibility criteria reflects the nascent stage of research in these specific domains, inherently limiting the generalizability and robustness of our findings.

Our analysis was based solely on the information reported in the full-text articles, which means that insufficient reporting in original studies could lead to incomplete data extraction or synthesis. Additionally, while our framework analysis of qualitative data, such as prompt strategies and reported outcomes, was systematic, it inherently involved a degree of subjective interpretation by the reviewers. Although consensus was reached through discussion to mitigate this, individual biases cannot be entirely eliminated.

This review specifically identified challenges inherent to LLM research that also serve as limitations to its own reproducibility. A significant proportion of the identified LLM-based CAs used proprietary models (eg, specific versions of GPT and Bard), which operate as “black boxes” with undisclosed architectures, training data, and update cycles. This means that replicating the exact behavior or outputs of these agents is inherently challenging, if not impossible, as the underlying model can change over time (“model drift”) even with the same prompt, representing a fundamental lack of version control and transparency within proprietary LLMs. Furthermore, while prompt engineering emerged as a critical design practice, our review found that prompts were often inconsistently documented or treated informally within the studies, impeding the ability of other researchers to precisely reproduce the designed agent behaviors or verify findings.

In conclusion, while this scoping review provides valuable insights into the emerging field of LLM-based CAs for well-being, these limitations should be considered when interpreting our findings and inform future research endeavors aiming for greater methodological rigor and transparency in this rapidly evolving domain.

### Future Works and Research Directions

This scoping review has systematically mapped the current landscape of LLM-based CAs for PA and cognitive training, identifying several critical gaps and promising avenues for future investigation. Building upon our findings, we propose the following key directions for future research.

#### Advancing LLM-Based CA Applications and Design

Given the nascent and exploratory nature of LLM-based CAs in cognitive training, future work should prioritize the development and rigorous evaluation of interventions grounded in validated cognitive protocols. This includes exploring their utility for specific cognitive functions beyond reminiscence and task scaffolding, such as executive functions or attention, potentially in clinical populations. Our review also highlighted a predominant focus on dyadic, one-to-one interactions, with “coaching’' being a commonly adopted role. Future research should thus investigate more diverse and complex social dynamics, explicitly exploring social setups such as companionship, alongside multiuser interfaces, group-based interventions, or integrating LLM-based CAs into broader community support systems for collective well-being. Furthermore, the observed fragility of LLM-based CA social roles underscores the need for designing systems with persistent memory mechanisms, state awareness, and adaptive logical layers. Research should explore how these structural reinforcements can enable more consistent, trustworthy, and long-term therapeutic alliances or human-agent relationships.

#### Strengthening Methodological Rigor and Transparency

There is a critical need for more robust quantitative evaluation of LLM-based CA effectiveness. Future studies should move beyond perceived usefulness and user satisfaction, using validated behavioral and cognitive outcome measures. This necessitates the adoption of mixed methods designs, ideally with control groups and longitudinal follow-up, to ascertain sustained impact. Our findings also underscore that prompt engineering is a critical, yet inconsistently documented, design practice. Future research should prioritize developing and adopting standardized methodologies for prompt design, iteration, and evaluation. Furthermore, the systematic and open sharing of detailed prompt structures, including parameters such as temperature or top-p, is essential for enabling the reproducibility of LLM-based CA behaviors, as suggested by the LLM guidelines [70] project initiated by Wagner et al [71]. Addressing reproducibility with proprietary LLMs is another fundamental challenge, as their inherent variability and black box nature (eg, model drift over time) present a significant impediment to replication. Future research must explicitly acknowledge these limitations, and when proprietary models are used, authors should meticulously document the exact model version, application programming interface details, and precise dates of interaction. The community should also explore and contribute to research using open-source LLMs, where version control and long-term reproducibility can be more readily ensured. Finally, while some studies share data, practices remain inconsistent. Future work should fully embrace comprehensive data sharing, adhering to FAIR principles. Specifically, for LLM-based interventions, this mandates a focus on more detailed and structured prompt documentation, enabling their precise reproduction for verification and future research.

### Conclusions

This scoping review meticulously charted the nascent field of LLM-based CAs in PA and cognitive training. Our synthesis revealed a dynamic yet underexplored landscape, marked by a strong emphasis on PA coaching and a reliance on dyadic, prompt-driven interactions. While these agents demonstrate initial promise in engagement and perceived usefulness, a critical evaluation points to significant gaps in methodological rigor and comprehensive outcome assessment. Crucially, the unique challenges posed by proprietary LLMs and the current lack of structured prompt sharing emerge as fundamental impediments to reproducibility in this rapidly evolving domain. Addressing these issues through rigorous evaluation and a strong commitment to open science will be paramount to advancing the scientific understanding and responsible deployment of LLM-based CAs for well-being.

## Supplementary material

10.2196/80123 Multimedia Appendix 1 The comprehensive, open-access materials necessary to ensure the transparency, reproducibility, and verifiability of the scoping review.

10.2196/80123 Checklist 1 PRISMA-ScR checklist.

## Abbreviations

AI: artificial intelligence
CA: conversational agent
FAIR: findable, accessible, interoperable, reusable
HCI: human-computer interaction
LLM: large language model
PA: physical activity
PRISMA-ScR: Preferred Reporting Items for Systematic Reviews and Meta-Analyses extension for Scoping Reviews
RQ: research question
WoS: Clarivate Web of Science
