# Supplementary material for: Large Language Model–Based Agents for Physical Activity and Cognitive Training: Scoping Review
Source: JMIR AI. 2026 Mar 12;5:e80123. doi: 10.2196/80123 (PMC12981376; doi:10.2196/80123)
Supplement: Multimedia Appendix 1 [file ai-v5-e80123-s001.zip › supplementary_materials_large_language_models_pa_ct_scoping_review/04_data_extraction/041_data_extraction_instructions.pdf]

Supplementary Material: Data Collection Instructions

Introduction

This document outlines the instructions for data extraction in a scoping review focused on studies involving large language model (LLM)-based conversational agents (CAs). The goal of this review is to systematically collect and analyze data to understand the design, implementation, and outcomes of these systems across various applications. By adhering to the guidelines provided, reviewers will ensure consistency and accuracy in capturing essential study characteristics, facilitating a comprehensive synthesis of the evidence.

Study purpose

Instruction: Identify and record the primary aim or research questions of the study.

Examples: "To evaluate the efficacy of an LLM-based conversational agent in healthcare" or "To develop a framework for LLM fine-tuning in educational settings."

LLM model

Instruction: Specify the name or type of large language model used in the study.

Examples: (e.g., GPT-3, BERT, BLOOM).

LLM model accessibility

Instruction: Indicate whether the LLM is open-source, proprietary, or has restricted access. If the model is accessible, insert the link to the model's weights.

Examples: "Open-source, + link to the model's weights" "Licensed," or "Custom-developed."

LLM fine-tuning

Instruction: Note if the LLM was fine-tuned for the study's purpose and describe the fine-tuning process briefly. If it is the case, check if the dataset is provided by the study (e.g., in the supplementary materials, or a git repository).

Examples: "Fine-tuned for mental health applications using a dataset of 10,000 conversations."

CA name

Instruction: Record the specific name of the conversational agent, if provided.

Examples: ELIZA, Wysa, etc.

CA form

Instruction: Describe the form of the conversational agent: text-based, voice-based, multimodal, or other formats.

CA role

Instruction: Define the role the conversational agent plays in the study.

Examples: "Personal coach", "Customer support agent", "Medical assistant", or “Companion”.

CA purpose

Instruction: Identify the main purpose of the conversational agent.

Examples: "To improve user engagement", "To provide physical activity recommendations", or "To support cognitive function"

Deployment

Instruction: Specify where and how the conversational agent was deployed (e.g., mobile app, web-based platform, or standalone software).

CA design characteristics

Instruction: Record notable design features of the conversational agent, such as user interface design, interaction style, or personalization capabilities.

Interactional structure

Instruction: Define whether the conversation involves a single user and the agent (dyadic) or multiple users interacting simultaneously (polyadic).

Software name

Instruction: Note the name of the software used for running or supporting the conversational agent, if applicable.

Cognitive training type

Instruction: Categorize the type of cognitive training being used or for which the system/study has been designed. Usually values are like: *dual-task, multi-domain, single-domain, hybrid, etc.*

Physical activity type

Instruction: Identify and record the specific type of activity mentioned (e.g., aerobic exercise, strength training, walking, yoga). If multiple activities are included, list all relevant types.

Prompt

Instruction: Identify and record the type of prompts used (e.g., open-ended, guided, multiple-choice, predefined templates). If available, note any details on prompt structure, customization, or adaptation based on user input.

Results

Instruction: Summarize the key findings or outcomes of the study as reported by the authors.

Examples: "The conversational agent improved user engagement by 20%", "The system was 90% accurate in identifying user intent."
