# Supplementary material for: Large Language Model–Based Agents for Physical Activity and Cognitive Training: Scoping Review
Source: JMIR AI. 2026 Mar 12;5:e80123. doi: 10.2196/80123 (PMC12981376; doi:10.2196/80123)
Supplement: Multimedia Appendix 1 [file ai-v5-e80123-s001.zip › supplementary_materials_large_language_models_pa_ct_scoping_review/03_screening/032_eligibility_criteria.pdf]

Supplementary Material - Large Language Model-Based Agents for Physical Activity and Cognitive Training: A Scoping Review: Eligibility Criteria

Introduction

Following best practices in research transparency and reproducibility, and in accordance with the PRISMA-ScR framework, this document outlines the eligibility criteria used to screen studies included in the review Large Language Model-Based Agents for Physical Activity and Cognitive Training: A Scoping Review. The criteria were developed based on prior literature and methodological guidance on scoping reviews. A full rationale and discussion of their application are provided in the main article.

Criteria

Articles following this set of rules will be deemed eligible for inclusion in the review:

a) Study involves the use of an LLM-based agent
b) Study involves humans from all age ranges and independently from their health status.
c) The study presents a computer program capable of simulating two-way human conversation, intended for purposes such as physical activity support, cognitive training. The program utilizes language (speech or text) and may incorporate non-language modalities, regardless of the input and output options.
d) The study encompasses the design, development, evaluation, or implementation of conversational agents, irrespective of the involvement of human users or the specific study design.
e) If the study is a cognitive intervention it has to address cognitive training.
f) The article has been published in a peer-reviewed international outlet conference or journal.

On the contrary, works not following this set of criteria will be deemed ineligible for inclusion in the review:

a) Full-text is not available
b) Articles written in languages other than English
c) Agents whose agency is not derived from the use of an LLM (e.g., relying on RASA, Google DialogFlow, etc.)
d) Commentaries, opinion papers, position papers, study protocols, or any article not presenting primary research
e) Conference abstracts
f) Books and books chapter
g) If the study is a cognitive intervention, it should not be about cognitive behavioral therapy, cognitive assistance, or cognitive well-being.
