# Supplementary material for: Large Language Model–Based Agents for Physical Activity and Cognitive Training: Scoping Review
Source: JMIR AI. 2026 Mar 12;5:e80123. doi: 10.2196/80123 (PMC12981376; doi:10.2196/80123)
Supplement: Multimedia Appendix 1 [file ai-v5-e80123-s001.zip › supplementary_materials_large_language_models_pa_ct_scoping_review/02_queries/021_database_queries_manual.pdf]

Supplementary Material: Detailed Database Search

Introduction

This document provides the complete and exact search queries executed for the scoping review, "Large Language Model-Based Agents for Physical Activity and Cognitive Training: A Scoping Review." Adhering to the FAIR principles (Findable, Accessible, Interoperable, Reusable) and enhancing the transparency and reproducibility of our methodology, this supplementary material details the precise search strategies employed across the selected databases.

Contents

This document includes:

- **Database-Specific Queries:** The full query strings for each of the two primary searches (K1 AND K2 AND K3; K1 AND K2 AND K4) as executed on Elsevier Scopus and Clarivate Web of Science.
- **Date Ranges:** Confirmation that all searches were restricted to publications from January 2018 to December 2024.
- **Direct Links (where applicable):** For Clarivate Web of Science, direct links to the executed search summaries are provided to facilitate immediate verification.

Queries

Elsevier Scopus requests

K1 AND K2 AND K3

```
TITLE-ABS-KEY("animated character*" OR "artificial agent*" OR "artificial intelligence assistant*" OR "assistive social agent*" OR "communicative agent*" OR "conversational agent*" OR "companion agent" OR "companion assistant*" OR "conversational agent*" OR "conversational assistant*" OR "digital assistant*" OR "embodied agent*" OR "interactive agent*" OR "interface agent*" OR "pedagogical agent*" OR "ECA" OR "relational agent*" OR "relational assistant*" OR "virtual agent*" OR "virtual assistant*" OR "virtual character" OR "virtual coach*" OR "virtual counselor*" OR "virtual health counselor*" OR "virtual health agent*" OR "virtual health coach" OR "virtual human" OR "virtual therapist*" OR "virtual advisor" OR "artificial companion*" OR "coaching system" OR "chatbot" OR "communication robot" OR "virtual expert" OR "virtual friend" OR "virtual tutor" OR "virtual instructor" OR "virtual personal trainer" OR "virtual companion*" OR "virtual carer" OR "virtual mentor" OR "e-coach*" OR "ecoach*" OR "virtual coach" OR "digital coach*" OR "digital avatar" OR "digital agent*") AND TITLE-ABS-KEY("Large Language Model*" OR "LLM*" OR "BERT" OR "ChatGPT" OR "GPT*" OR "Llama" OR "Claude" OR "Gemini" OR "Bard" OR "Falcon" OR "BART" OR "PaLM" OR "Mistral" OR "Mixtral" OR "Bloom" OR "Phi" OR "pretrained model" OR "transformer*" OR "generative AI" OR "genAI") AND TITLE-ABS-KEY("physical activity" OR "physical* activ*" OR "sport*" OR "physical exercis*" OR "workout" OR "exergam*" OR "fitness gam*" OR "digital play" OR "physical action gam*" OR "health gam*" OR "physical fitness" OR "physical education" OR "motor activity" OR "walking" OR "running" OR "cycling" OR "swimming" OR "hiking" OR "leisure activity") AND PUBYEAR > 2017 AND PUBYEAR < 2025
```

K1 AND K2 AND K4

TITLE-ABS-KEY("animated character*" OR "artificial agent*" OR "artificial intelligence assistant*" OR "assistive social agent*" OR "communicative agent*" OR "conversational agent*" OR "companion agent" OR "companion assistant*" OR "conversational agent*" OR "conversational assistant*" OR "digital assistant*" OR "embodied agent*" OR "interactive agent*" OR "interface agent*" OR "pedagogical agent*" OR "ECA" OR "relational agent*" OR "relational assistant*" OR "virtual agent*" OR "virtual assistant*" OR "virtual character" OR "virtual coach*" OR "virtual counselor*" OR "virtual health counselor*" OR "virtual health agent*" OR "virtual health coach" OR "virtual human" OR "virtual therapist*" OR "virtual advisor" OR "artificial companion*" OR "coaching system" OR "chatbot" OR "communication robot" OR "virtual expert" OR "virtual friend" OR "virtual tutor" OR "virtual instructor" OR "virtual personal trainer" OR "virtual companion*" OR "virtual carer" OR "virtual mentor" OR "e-coach*" OR "ecoach*" OR "virtual coach" OR "digital coach*" OR "digital avatar" OR "digital agent*") AND TITLE-ABS-KEY("Large Language Model*" OR "LLM*" OR "BERT" OR "ChatGPT" OR "GPT*" OR "Llama" OR "Claude" OR "Gemini" OR "Bard" OR "Falcon" OR "BART" OR "PaLM" OR "Mistral" OR "Mixtral" OR "Bloom" OR "Phi" OR "pretrained model" OR "transformer*" OR "generative AI" OR "genAI") AND TITLE-ABS-KEY("executive function*" OR "cognitive load" OR "cognitive train*" OR "cognitive therapy" OR "cognitive intervention" OR "memory train*" OR "cognitive impair*" OR "dementia" OR "Alzheimer" OR "MCI" OR "cognitive decline" OR "cognitive disorder*" OR "cognitive deficit*" OR "brain train*" OR "brain workout" OR "brain exercis*" OR "cognit* retrain*") AND PUBYEAR > 2017 AND PUBYEAR < 2025

Clarivate Web of Science

K1 AND K2 AND K3

TS=("animated character*" OR "artificial agent*" OR "artificial intelligence assistant*" OR "assistive social agent*" OR "communicative agent*" OR "conversational agent*" OR "companion agent" OR "companion assistant*" OR "conversational agent*" OR "conversational assistant*" OR "digital assistant*" OR "embodied agent*" OR "interactive agent*" OR "interface agent*" OR "pedagogical agent*" OR "ECA" OR "relational agent*" OR "relational assistant*" OR "virtual agent*" OR "virtual assistant*" OR "virtual character" OR "virtual coach*" OR "virtual counselor*" OR "virtual health counselor*" OR "virtual health agent*" OR "virtual health coach" OR "virtual human" OR "virtual therapist*" OR "virtual advisor" OR "artificial companion*" OR "coaching system" OR "chatbot" OR "communication robot" OR "virtual expert" OR "virtual friend" OR "virtual tutor" OR "virtual instructor" OR "virtual personal trainer" OR "virtual companion*" OR "virtual carer" OR "virtual mentor" OR "e-coach*" OR "ecoach*" OR "virtual coach" OR "digital coach*" OR "digital avatar" OR "digital agent*") AND TS=("Large Language Model*" OR "LLM*" OR "BERT" OR "ChatGPT" OR "GPT*" OR "Llama" OR "Claude" OR "Gemini" OR "Bard" OR "Falcon" OR "BART" OR "PaLM" OR "Mistral" OR "Mixtral" OR "Bloom" OR "Phi" OR "pretrained model" OR "transformer*" OR "generative AI" OR "genAI") AND TS=("physical activity" OR "physical* activ*" OR "sport*" OR "physical exercis*" OR "workout" OR "exergam*" OR "fitness gam*" OR "digital play" OR "physical action gam*" OR "health gam*" OR "physical fitness" OR "physical education" OR "motor activity" OR "walking" OR "running" OR "cycling" OR "swimming" OR "hiking" OR "leisure activity")

Index Date 2018-01-01 to 2024-12-31

Full request is accessible using the following link: https://www.webofscience.com/wos/woscc/summary/baed2932-d78a-4802-9b55-765f6197e4bb-0180cf0b2f/relevance/1, last accessed October 2025

K1 AND K2 AND K4

TS=("animated character*" OR "artificial agent*" OR "artificial intelligence assistant*" OR "assistive social agent*" OR "communicative agent*" OR "conversational agent*" OR "companion agent" OR "companion assistant*" OR "conversational agent*" OR "conversational assistant*" OR "digital assistant*" OR "embodied agent*" OR "interactive agent*" OR "interface agent*" OR "pedagogical agent*" OR "ECA" OR "relational agent*" OR "relational assistant*" OR "virtual agent*" OR "virtual assistant*" OR "virtual character" OR "virtual coach*" OR "virtual counselor*" OR "virtual health counselor*" OR "virtual health agent*" OR "virtual health coach" OR "virtual human" OR "virtual therapist*" OR "virtual advisor" OR "artificial companion*" OR "coaching system" OR "chatbot" OR "communication robot" OR "virtual expert" OR "virtual friend" OR "virtual tutor" OR "virtual instructor" OR "virtual personal trainer" OR "virtual companion*" OR "virtual carer" OR "virtual mentor" OR "e-coach*" OR "ecoach*" OR "virtual coach" OR "digital coach*" OR "digital avatar" OR "digital agent*") AND TS=("Large Language Model*" OR "LLM*" OR "BERT" OR "ChatGPT" OR "GPT*" OR "Llama" OR "Claude" OR "Gemini" OR "Bard" OR "Falcon" OR "BART" OR "PaLM" OR "Mistral" OR "Mixtral" OR "Bloom" OR "Phi" OR "pretrained model" OR "transformer*" OR "generative AI" OR "genAI") AND TS=("executive function*" OR "cognitive load" OR "cognitive train*" OR "cognitive therapy" OR "cognitive intervention" OR

"memory train*" OR "cognitive impair*" OR "dementia" OR "Alzheimer" OR "MCI" OR "cognitive decline" OR "cognitive disorder*" OR "cognitive deficit*" OR "brain train*" OR "brain workout" OR "brain exercis*" OR "cognit* retrain*")

Index Date 2018-01-01 to 2024-12-31
Full request is accessible using the following link:
https://www.webofscience.com/wos/woscc/summary/2398cee4-5d1d-499f-a980-de5b848340fb-0180cf670a/relevance/1, last accessed October 2025

IEEE Xplore

K1 AND K2 AND K3

Title

("Publication Title":"animated character" OR "Publication Title":"artificial agent" OR "Publication Title":"artificial intelligence assistant" OR "Publication Title":"assistive social agent" OR "Publication Title": "communicative agent" OR "Publication Title":"conversational agent" OR "Publication Title":"companion agent" OR "Publication Title": "companion assistant" OR "Publication Title":"conversational agent" OR "Publication Title": "conversational assistant" OR "Publication Title": "digital assistant" OR "Publication Title": "embodied agent" OR "Publication Title":"interactive agent" OR "Publication Title":"interface agent" OR "Publication Title":"pedagogical agent" OR "Publication Title":"ECA" OR "Publication Title":"relational agent" OR "Publication Title":"relational assistant" OR "Publication Title":"virtual agent" OR "Publication Title":"virtual assistant" OR "Publication Title": "virtual character" OR "Publication Title":"virtual coach" OR "Publication Title":"virtual counselor" OR "Publication Title": "virtual health counselor" OR "Publication Title":"virtual health agent" OR "Publication Title": "virtual health coach" OR "Publication Title":"virtual human" OR "Publication Title":"virtual therapist" OR "Publication Title":"virtual advisor" OR "Publication Title":"artificial companion" OR "Publication Title":"coaching system" OR "Publication Title":"chatbot" OR "Publication Title":"communication robot" OR "Publication Title":"virtual expert" OR "Publication Title":"virtual friend" OR "Publication Title":"virtual tutor" OR "Publication Title":"virtual instructor" OR "Publication Title":"virtual personal trainer" OR "Publication Title":"virtual companion" OR "Publication Title":"virtual carer" OR "Publication Title":"virtual mentor" OR "Publication Title":"e-coach" OR "Publication Title":"ecoach" OR "Publication Title":"virtual coach" OR "Publication Title":"digital coach" OR "Publication Title":"digital avatar" OR "Publication Title":"digital agent") AND ("Publication Title":"Large Language Model" OR "Publication Title":"LLM" OR "Publication Title":"BERT" OR "Publication Title":"ChatGPT" OR "Publication Title":"GPT" OR "Publication Title":"Llama" OR "Publication Title":"Claude" OR "Publication Title":"Gemini" OR "Publication Title":"Bard" OR "Publication Title":"Falcon" OR "Publication Title":"BART" OR "Publication Title":"PaLM" OR "Publication Title":"Mistral" OR "Publication Title":"Mixtral" OR "Publication Title":"Bloom" OR "Publication Title":"Phi", "Publication Title":"pretrained model", "Publication Title":"transformer*", "Publication Title":"generative AI", "Publication Title": "genAI") AND ("Publication Title":"physical activity" OR "Publication Title":"physical* activ*" OR "Publication Title":"sport*" OR "Publication Title":"physical exercis*" OR "Publication Title":"workout" OR "Publication Title":"exergam*" OR "Publication Title":"fitness gam*" OR "Publication Title":"digital play" OR "Publication Title":"physical action gam*" OR "Publication Title":"health gam*" OR "Publication Title":"physical fitness" OR "Publication Title":"physical education" OR "Publication Title":"motor activity" OR "Publication Title":"walking" OR "Publication Title":"running" OR "Publication Title":"cycling" OR "Publication Title":"swimming" OR "Publication Title":"hiking" OR "Publication Title":"leisure activity")

Abstract

("Abstract":"animated character" OR "Abstract":"artificial agent" OR "Abstract":"artificial intelligence assistant" OR "Abstract":"assistive social agent" OR "Abstract": "communicative agent" OR "Abstract":"conversational agent" OR "Abstract":"companion agent" OR "Abstract": "companion assistant" OR "Abstract":"conversational agent" OR "Abstract": "conversational assistant" OR "Abstract": "digital assistant" OR "Abstract": "embodied agent" OR "Abstract":"interactive agent" OR "Abstract":"interface agent" OR "Abstract":"pedagogical agent" OR "Abstract":"ECA" OR "Abstract":"relational agent" OR "Abstract":"relational assistant" OR "Abstract":"virtual agent" OR "Abstract":"virtual assistant" OR "Abstract": "virtual character" OR "Abstract":"virtual coach" OR "Abstract":"virtual counselor" OR "Abstract": "virtual health counselor" OR "Abstract":"virtual health agent" OR "Abstract": "virtual health coach" OR "Abstract":"virtual human" OR "Abstract":"virtual therapist" OR "Abstract":"virtual advisor" OR "Abstract":"artificial companion" OR "Abstract":"coaching system" OR "Abstract":"chatbot" OR "Abstract":"communication robot" OR "Abstract":"virtual expert" OR "Abstract":"virtual friend" OR "Abstract":"virtual tutor" OR "Abstract":"virtual instructor" OR "Abstract":"virtual personal trainer" OR "Abstract":"virtual companion" OR "Abstract":"virtual carer" OR "Abstract":"virtual mentor" OR "Abstract":"e-coach" OR "Abstract":"ecoach" OR "Abstract":"virtual coach" OR "Abstract":"digital coach" OR "Abstract":"digital avatar" OR "Abstract":"digital agent") AND ("Abstract":"Large Language Model" OR "Abstract":"LLM" OR "Abstract":"BERT" OR "Abstract":"ChatGPT" OR "Abstract":"GPT" OR "Abstract":"Llama" OR "Abstract":"Claude" OR "Abstract":"Gemini" OR "Abstract":"Bard" OR "Abstract":"Falcon" OR "Abstract":"BART" OR "Abstract":"PaLM" OR "Abstract":"Mistral" OR "Abstract":"Mixtral" OR "Abstract":"Bloom" OR "Abstract":"Phi", "Abstract":"pretrained model", "Abstract":"transformer*", "Abstract":"generative AI", "Abstract": "genAI") AND ("Abstract":"physical activity" OR "Abstract":"physical* activ*" OR "Abstract":"sport*" OR "Abstract":"physical exercis*" OR "Abstract":"workout" OR "Abstract":"exergam*" OR "Abstract":"fitness gam*" OR "Abstract":"digital play" OR "Abstract":"physical action gam*" OR "Abstract":"health gam*" OR "Abstract":"physical fitness" OR "Abstract":"physical education" OR "Abstract":"motor activity" OR "Abstract":"walking" OR "Abstract":"running" OR "Abstract":"cycling" OR "Abstract":"swimming" OR "Abstract":"hiking" OR "Abstract":"leisure activity")

Keywords

("Author Keywords":"animated character" OR "Author Keywords":"artificial agent" OR "Author Keywords":"artificial intelligence assistant" OR "Author Keywords":"assistive social agent" OR "Author Keywords": "communicative agent" OR "Author Keywords":"conversational agent" OR "Author Keywords":"companion agent" OR "Author Keywords": "companion assistant" OR "Author Keywords":"conversational agent" OR "Author Keywords": "conversational assistant" OR "Author Keywords": "digital assistant" OR "Author Keywords": "embodied agent" OR "Author Keywords":"interactive agent" OR "Author Keywords":"interface agent" OR "Author Keywords":"pedagogical agent" OR "Author Keywords":"ECA" OR "Author Keywords":"relational agent" OR "Author Keywords":"relational assistant" OR "Author Keywords":"virtual agent" OR "Author Keywords":"virtual assistant" OR "Author Keywords": "virtual character" OR "Author Keywords":"virtual coach" OR "Author Keywords":"virtual counselor" OR "Author Keywords": "virtual health counselor" OR "Author Keywords":"virtual health agent" OR "Author Keywords": "virtual health coach" OR "Author Keywords":"virtual human" OR "Author Keywords":"virtual therapist" OR "Author Keywords":"virtual advisor" OR "Author Keywords":"artificial companion" OR "Author Keywords":"coaching system" OR "Author Keywords":"chatbot" OR "Author Keywords":"communication robot" OR "Author Keywords":"virtual expert" OR "Author Keywords":"virtual friend" OR "Author Keywords":"virtual tutor" OR "Author Keywords":"virtual instructor" OR "Author Keywords":"virtual personal trainer" OR "Author Keywords":"virtual companion" OR "Author Keywords":"virtual carer" OR "Author Keywords":"virtual mentor" OR "Author Keywords":"e-coach" OR "Author Keywords":"ecoach" OR "Author Keywords":"virtual coach" OR "Author Keywords":"digital coach" OR "Author Keywords":"digital avatar" OR "Author Keywords":"digital agent") AND ("Author Keywords":"Large Language Model" OR "Author Keywords":"LLM" OR "Author Keywords":"BERT" OR "Author Keywords":"ChatGPT" OR "Author Keywords":"GPT" OR "Author Keywords":"Llama" OR "Author Keywords":"Claude" OR "Author Keywords":"Gemini" OR "Author Keywords":"Bard" OR "Author Keywords":"Falcon" OR "Author Keywords":"BART" OR "Author Keywords":"PaLM" OR "Author Keywords":"Mistral" OR "Author Keywords":"Mixtral" OR "Author Keywords":"Bloom" OR "Author Keywords":"Phi", "Author Keywords":"pretrained model", "Author Keywords":"transformer*", "Author Keywords":"generative AI", "Author Keywords": "genAI") AND ("Author Keywords":"physical activity" OR "Author Keywords":"physical* activ*" OR "Author Keywords":"sport*" OR "Author Keywords":"physical exercis*" OR "Author Keywords":"workout" OR "Author Keywords":"exergam*" OR "Author Keywords":"fitness gam*" OR "Author Keywords":"digital play" OR "Author Keywords":"physical action gam*" OR "Author Keywords":"health gam*" OR "Author Keywords":"physical fitness" OR "Author Keywords":"physical education" OR "Author Keywords":"motor activity" OR "Author Keywords":"walking" OR "Author Keywords":"running" OR "Author Keywords":"cycling" OR "Author Keywords":"swimming" OR "Author Keywords":"hiking" OR "Author Keywords":"leisure activity")

K1 AND K2 AND K4

Title

("Publication Title":"animated character" OR "Publication Title":"artificial agent" OR "Publication Title":"artificial intelligence assistant" OR "Publication Title":"assistive social agent" OR "Publication Title": "communicative agent" OR "Publication Title":"conversational agent" OR "Publication Title":"companion agent" OR "Publication Title": "companion assistant" OR "Publication Title":"conversational agent" OR "Publication Title": "conversational assistant" OR "Publication Title": "digital assistant" OR "Publication Title": "embodied agent" OR "Publication Title":"interactive agent" OR "Publication Title":"interface agent" OR "Publication Title":"pedagogical agent" OR "Publication Title":"ECA" OR "Publication Title":"relational agent" OR "Publication Title":"relational assistant" OR "Publication Title":"virtual agent" OR "Publication Title":"virtual assistant" OR "Publication Title": "virtual character" OR "Publication Title":"virtual coach" OR "Publication Title":"virtual counselor" OR "Publication Title": "virtual health counselor" OR "Publication Title":"virtual health agent" OR "Publication Title": "virtual health coach" OR "Publication Title":"virtual human" OR "Publication Title":"virtual therapist" OR "Publication Title":"virtual advisor" OR "Publication Title":"artificial companion" OR "Publication Title":"coaching system" OR "Publication Title":"chatbot" OR "Publication Title":"communication robot" OR "Publication Title":"virtual expert" OR "Publication Title":"virtual friend" OR "Publication Title":"virtual tutor" OR "Publication Title":"virtual instructor" OR "Publication Title":"virtual personal trainer" OR "Publication Title":"virtual companion" OR "Publication Title":"virtual carer" OR "Publication Title":"virtual mentor" OR "Publication Title":"e-coach" OR "Publication Title":"ecoach" OR "Publication Title":"virtual coach" OR "Publication Title":"digital coach" OR "Publication Title":"digital avatar" OR "Publication Title":"digital agent") AND ("Publication Title":"Large Language Model" OR "Publication Title":"LLM" OR "Publication Title":"BERT" OR "Publication Title":"ChatGPT" OR "Publication Title":"GPT" OR "Publication Title":"Llama" OR "Publication Title":"Claude" OR "Publication Title":"Gemini" OR "Publication Title":"Bard" OR "Publication Title":"Falcon" OR "Publication Title":"BART" OR "Publication Title":"PaLM" OR "Publication Title":"Mistral" OR "Publication Title":"Mixtral" OR "Publication Title":"Bloom" OR "Publication Title":"Phi", "Publication Title":"pretrained model", "Publication Title":"transformer*", "Publication Title":"generative AI", "Publication Title": "genAI") AND ("Publication Title":"executive function*" OR "Publication Title":"cognitive load" OR "Publication Title":"cognitive train*" OR "Publication Title":"cognitive therapy" OR "Publication Title":"cognitive intervention" OR "Publication Title":"memory train*" OR "Publication Title": "cognitive impair*" OR "Publication Title":"dementia" OR "Publication Title":"Alzheimer" OR "Publication Title":"MCI" OR "Publication Title":"cognitive decline" OR "Publication Title":"cognitive disorder*" OR "Publication Title":"cognitive deficit*" OR "Publication Title":"brain train*" OR "Publication Title":"brain workout" OR "Publication Title": "brain exercis*" OR "Publication Title":"cognit* retrain*")

Abstract

("Abstract":"animated character" OR "Abstract":"artificial agent" OR "Abstract":"artificial intelligence assistant" OR "Abstract":"assistive social agent" OR "Abstract": "communicative agent" OR "Abstract":"conversational agent" OR "Abstract":"companion agent" OR "Abstract": "companion assistant" OR "Abstract":"conversational agent" OR "Abstract": "conversational assistant" OR "Abstract": "digital assistant" OR "Abstract": "embodied agent" OR "Abstract":"interactive agent" OR "Abstract":"interface agent" OR "Abstract":"pedagogical agent" OR "Abstract":"ECA" OR "Abstract":"relational agent" OR "Abstract":"relational assistant" OR "Abstract":"virtual agent" OR "Abstract":"virtual assistant" OR "Abstract": "virtual character" OR "Abstract":"virtual coach" OR "Abstract":"virtual counselor" OR "Abstract": "virtual health counselor" OR "Abstract":"virtual health agent" OR "Abstract": "virtual health coach" OR "Abstract":"virtual human" OR "Abstract":"virtual therapist" OR "Abstract":"virtual advisor" OR "Abstract":"artificial companion" OR "Abstract":"coaching system" OR "Abstract":"chatbot" OR "Abstract":"communication robot" OR "Abstract":"virtual expert" OR "Abstract":"virtual friend" OR "Abstract":"virtual tutor" OR "Abstract":"virtual instructor" OR "Abstract":"virtual personal trainer" OR "Abstract":"virtual companion" OR "Abstract":"virtual carer" OR "Abstract":"virtual mentor" OR "Abstract":"e-coach" OR "Abstract":"ecoach" OR "Abstract":"virtual coach" OR "Abstract":"digital coach" OR "Abstract":"digital avatar" OR "Abstract":"digital agent") AND ("Abstract":"Large Language Model" OR "Abstract":"LLM" OR "Abstract":"BERT" OR "Abstract":"ChatGPT" OR "Abstract":"GPT" OR "Abstract":"Llama" OR "Abstract":"Claude" OR "Abstract":"Gemini" OR "Abstract":"Bard" OR "Abstract":"Falcon" OR "Abstract":"BART" OR "Abstract":"PaLM" OR "Abstract":"Mistral" OR "Abstract":"Mixtral" OR "Abstract":"Bloom" OR "Abstract":"Phi", "Abstract":"pretrained model", "Abstract":"transformer*", "Abstract":"generative AI", "Abstract": "genAI") AND ("Abstract":"executive function*" OR "Abstract":"cognitive load" OR "Abstract":"cognitive train*" OR "Abstract":"cognitive therapy" OR "Abstract":"cognitive intervention" OR "Abstract":"memory train*" OR "Abstract": "cognitive impair*" OR "Abstract":"dementia" OR "Abstract":"Alzheimer" OR "Abstract":"MCI" OR "Abstract":"cognitive decline" OR "Abstract":"cognitive disorder*" OR "Abstract":"cognitive deficit*" OR "Abstract":"brain train*" OR "Abstract":"brain workout" OR "Abstract": "brain exercis*" OR "Abstract":"cognit* retrain*")

Keywords

("Author Keywords":"animated character" OR "Author Keywords":"artificial agent" OR "Author Keywords":"artificial intelligence assistant" OR "Author Keywords":"assistive social agent" OR "Author Keywords": "communicative agent" OR "Author Keywords":"conversational agent" OR "Author Keywords":"companion agent" OR "Author Keywords": "companion assistant" OR "Author Keywords":"conversational agent" OR "Author Keywords": "conversational assistant" OR "Author Keywords": "digital assistant" OR "Author Keywords": "embodied agent" OR "Author Keywords":"interactive agent" OR "Author Keywords":"interface agent" OR "Author Keywords":"pedagogical agent" OR "Author Keywords":"ECA" OR "Author Keywords":"relational agent" OR "Author Keywords":"relational assistant" OR "Author Keywords":"virtual agent" OR "Author Keywords":"virtual assistant" OR "Author Keywords": "virtual character" OR "Author Keywords":"virtual coach" OR "Author Keywords":"virtual counselor" OR "Author Keywords": "virtual health counselor" OR "Author Keywords":"virtual health agent" OR "Author Keywords": "virtual health coach" OR "Author Keywords":"virtual human" OR "Author Keywords":"virtual therapist" OR "Author Keywords":"virtual advisor" OR "Author Keywords":"artificial companion" OR "Author Keywords":"coaching system" OR "Author Keywords":"chatbot" OR "Author Keywords":"communication robot" OR "Author Keywords":"virtual expert" OR "Author Keywords":"virtual friend" OR "Author Keywords":"virtual tutor" OR "Author Keywords":"virtual instructor" OR "Author Keywords":"virtual personal trainer" OR "Author Keywords":"virtual companion" OR "Author Keywords":"virtual carer" OR "Author Keywords":"virtual mentor" OR "Author Keywords":"e-coach" OR "Author Keywords":"ecoach" OR "Author Keywords":"virtual coach" OR "Author Keywords":"digital coach" OR "Author Keywords":"digital avatar" OR "Author Keywords":"digital agent") AND ("Author Keywords":"Large Language Model" OR "Author Keywords":"LLM" OR "Author Keywords":"BERT" OR "Author Keywords":"ChatGPT" OR "Author Keywords":"GPT" OR "Author Keywords":"Llama" OR "Author Keywords":"Claude" OR "Author Keywords":"Gemini" OR "Author Keywords":"Bard" OR "Author Keywords":"Falcon" OR "Author Keywords":"BART" OR "Author Keywords":"PaLM" OR "Author Keywords":"Mistral" OR "Author Keywords":"Mixtral" OR "Author Keywords":"Bloom" OR "Author Keywords":"Phi", "Author Keywords":"pretrained model", "Author Keywords":"transformer*", "Author Keywords":"generative AI", "Author Keywords": "genAI") AND ("Author Keywords":"executive function*" OR "Author Keywords":"cognitive load" OR "Author Keywords":"cognitive train*" OR "Author Keywords":"cognitive therapy" OR "Author Keywords":"cognitive intervention" OR "Author Keywords":"memory train*" OR "Author Keywords": "cognitive impair*" OR "Author Keywords":"dementia" OR "Author Keywords":"Alzheimer" OR "Author Keywords":"MCI" OR "Author Keywords":"cognitive decline" OR "Author Keywords":"cognitive disorder*" OR "Author Keywords":"cognitive deficit*" OR "Author Keywords":"brain train*" OR "Author Keywords":"brain workout" OR "Author Keywords": "brain exercis*" OR "Author Keywords":"cognit* retrain*")

ACM DL

K1 AND K2 AND K3

Title

[[Title: "animated character*"] OR [Title: "artificial agent*"] OR [Title: "artificial intelligence assistant*"] OR [Title: "assistive social agent*"] OR [Title: "communicative agent*"] OR [Title: "conversational agent*"] OR [Title: "companion agent"] OR [Title: "companion assistant*"] OR [Title: "conversational agent*"] OR [Title: "conversational assistant*"] OR [Title: "digital assistant*"] OR [Title: "embodied agent*"] OR [Title: "interactive agent*"] OR [Title: "interface agent*"] OR [Title: "pedagogical agent*"] OR [Title: "eca"] OR [Title: "relational agent*"] OR [Title: "relational assistant*"] OR [Title: "virtual agent*"] OR [Title: "virtual assistant*"] OR [Title: "virtual character"] OR [Title: "virtual coach*"] OR [Title: "virtual counselor*"] OR [Title: "virtual health counselor*"] OR [Title: "virtual health agent*"] OR [Title: "virtual health coach"] OR [Title: "virtual human"] OR [Title: "virtual therapist*"] OR [Title: "virtual advisor"] OR [Title: "artificial companion*"] OR [Title: "coaching system"] OR [Title: "chatbot"] OR [Title: "communication robot"] OR [Title: "virtual expert"] OR [Title: "virtual friend"] OR [Title: "virtual tutor"] OR [Title: "virtual instructor"] OR [Title: "virtual personal trainer"] OR [Title: "virtual companion*"] OR [Title: "virtual carer"] OR [Title: "virtual mentor"] OR [Title: "e-coach*"] OR [Title: "ecoach*"] OR [Title: "virtual coach"] OR [Title: "digital coach*"] OR [Title: "digital avatar"] OR [Title: "digital agent*"]] AND [[Title: "large language model*"] OR [Title: "llm*"] OR [Title: "bert"] OR [Title: "chatgpt"] OR [Title: "gpt*"] OR [Title: "llama"] OR [Title: "claude"] OR [Title: "gemini"] OR [Title: "bard"] OR [Title: "falcon"] OR [Title: "bart"] OR [Title: "palm"] OR [Title: "mistral"] OR [Title: "mixtral"] OR [Title: "bloom"] OR [Title: "phi"] OR [Title: "pretrained model"] OR [Title: "transformer*"] OR [Title: "generative ai"] OR [Title: "genai"]] AND [[Title: "physical activity"] OR [Title: "physical* activ*"] OR [Title: "sport*"] OR [Title: "physical exercis*"] OR [Title: "workout"] OR [Title: "exergam*"] OR [Title: "fitness gam*"] OR [Title: "digital play"] OR [Title: "physical action gam*"] OR [Title: "health gam*"] OR [Title: "physical fitness"] OR [Title: "physical education"] OR [Title:

"motor activity"] OR [Title: "walking"] OR [Title: "running"] OR [Title: "cycling"] OR [Title: "swimming"] OR [Title: "hiking"] OR [Title: "leisure activity"]] AND [E-Publication Date: (01/01/2018 TO 31/12/2024)]

Abstract

[[Abstract: "animated character*"] OR [Abstract: "artificial agent*"] OR [Abstract: "artificial intelligence assistant*"] OR [Abstract: "assistive social agent*"] OR [Abstract: "communicative agent*"] OR [Abstract: "conversational agent*"] OR [Abstract: "companion agent"] OR [Abstract: "companion assistant*"] OR [Abstract: "conversational agent*"] OR [Abstract: "conversational assistant*"] OR [Abstract: "digital assistant*"] OR [Abstract: "embodied agent*"] OR [Abstract: "interactive agent*"] OR [Abstract: "interface agent*"] OR [Abstract: "pedagogical agent*"] OR [Abstract: "eca"] OR [Abstract: "relational agent*"] OR [Abstract: "relational assistant*"] OR [Abstract: "virtual agent*"] OR [Abstract: "virtual assistant*"] OR [Abstract: "virtual character"] OR [Abstract: "virtual coach*"] OR [Abstract: "virtual counselor*"] OR [Abstract: "virtual health counselor*"] OR [Abstract: "virtual health agent*"] OR [Abstract: "virtual health coach"] OR [Abstract: "virtual human"] OR [Abstract: "virtual therapist*"] OR [Abstract: "virtual advisor"] OR [Abstract: "artificial companion*"] OR [Abstract: "coaching system"] OR [Abstract: "chatbot"] OR [Abstract: "communication robot"] OR [Abstract: "virtual expert"] OR [Abstract: "virtual friend"] OR [Abstract: "virtual tutor"] OR [Abstract: "virtual instructor"] OR [Abstract: "virtual personal trainer"] OR [Abstract: "virtual companion*"] OR [Abstract: "virtual carer"] OR [Abstract: "virtual mentor"] OR [Abstract: "e-coach*"] OR [Abstract: "ecoach*"] OR [Abstract: "virtual coach"] OR [Abstract: "digital coach*"] OR [Abstract: "digital avatar"] OR [Abstract: "digital agent*"]] AND [[Abstract: "large language model*"] OR [Abstract: "llm*"] OR [Abstract: "bert"] OR [Abstract: "chatgpt"] OR [Abstract: "gpt*"] OR [Abstract: "llama"] OR [Abstract: "claude"] OR [Abstract: "gemini"] OR [Abstract: "bard"] OR [Abstract: "falcon"] OR [Abstract: "bart"] OR [Abstract: "palm"] OR [Abstract: "mistral"] OR [Abstract: "mixtral"] OR [Abstract: "bloom"] OR [Abstract: "phi"] OR [Abstract: "pretrained model"] OR [Abstract: "transformer*"] OR [Abstract: "generative AI"] OR [Abstract: "genAI"]] AND [[Abstract: "physical activity"] OR [Abstract: "physical* activ*"] OR [Abstract: "sport*"] OR [Abstract: "physical exercis*"] OR [Abstract: "workout"] OR [Abstract: "exergam*"] OR [Abstract: "fitness gam*"] OR [Abstract: "digital play"] OR [Abstract: "physical action gam*"] OR [Abstract: "health gam*"] OR [Abstract: "physical fitness"] OR [Abstract: "physical education"] OR [Abstract: "motor activity"] OR [Abstract: "walking"] OR [Abstract: "running"] OR [Abstract: "cycling"] OR [Abstract: "swimming"] OR [Abstract: "hiking"] OR [Abstract: "leisure activity"]] AND [E-Publication Date: (01/01/2018 TO 12/31/2024)]

Keywords

[[Keywords: "animated character*"] OR [Keywords: "artificial agent*"] OR [Keywords: "artificial intelligence assistant*"] OR [Keywords: "assistive social agent*"] OR [Keywords: "communicative agent*"] OR [Keywords: "conversational agent*"] OR [Keywords: "companion agent"] OR [Keywords: "companion assistant*"] OR [Keywords: "conversational agent*"] OR [Keywords: "conversational assistant*"] OR [Keywords: "digital assistant*"] OR [Keywords: "embodied agent*"] OR [Keywords: "interactive agent*"] OR [Keywords: "interface agent*"] OR [Keywords: "pedagogical agent*"] OR [Keywords: "eca"] OR [Keywords: "relational agent*"] OR [Keywords: "relational assistant*"] OR [Keywords: "virtual agent*"] OR [Keywords: "virtual assistant*"] OR [Keywords: "virtual character"] OR [Keywords: "virtual coach*"] OR [Keywords: "virtual counselor*"] OR [Keywords: "virtual health counselor*"] OR [Keywords: "virtual health agent*"] OR [Keywords: "virtual health coach"] OR [Keywords: "virtual human"] OR [Keywords: "virtual therapist*"] OR [Keywords: "virtual advisor"] OR [Keywords: "artificial companion*"] OR [Keywords: "coaching system"] OR [Keywords: "chatbot"] OR [Keywords: "communication robot"] OR [Keywords: "virtual expert"] OR [Keywords: "virtual friend"] OR [Keywords: "virtual tutor"] OR [Keywords: "virtual instructor"] OR [Keywords: "virtual personal trainer"] OR [Keywords: "virtual companion*"] OR [Keywords: "virtual carer"] OR [Keywords: "virtual mentor"] OR [Keywords: "e-coach*"] OR [Keywords: "ecoach*"] OR [Keywords: "virtual coach"] OR [Keywords: "digital coach*"] OR [Keywords: "digital avatar"] OR [Keywords: "digital agent*"]] AND [[Keywords: "large language model*"] OR [Keywords: "llm*"] OR [Keywords: "bert"] OR [Keywords: "chatgpt"] OR [Keywords: "gpt*"] OR [Keywords: "llama"] OR [Keywords: "claude"] OR [Keywords: "gemini"] OR [Keywords: "bard"] OR [Keywords: "falcon"] OR [Keywords: "bart"] OR [Keywords: "palm"] OR [Keywords: "mistral"] OR [Keywords: "mixtral"] OR [Keywords: "bloom"] OR [Keywords: "phi"] OR [Keywords: "pretrained model"] OR [Keywords: "transformer*"] OR [Keywords: "generative ai"] OR [Keywords: "genai"]] AND [[Keywords: "physical activity"] OR [Keywords: "physical* activ*"] OR [Keywords: "sport*"] OR [Keywords: "physical exercis*"] OR [Keywords: "workout"] OR [Keywords: "exergam*"] OR [Keywords: "fitness gam*"] OR [Keywords: "digital play"] OR [Keywords: "physical action gam*"] OR [Keywords: "health gam*"] OR [Keywords: "physical fitness"] OR [Keywords: "physical education"] OR [Keywords: "motor activity"] OR [Keywords: "walking"] OR [Keywords: "running"] OR

[Keywords: "cycling"] OR [Keywords: "swimming"] OR [Keywords: "hiking"] OR [Keywords: "leisure activity"]] AND [E-Publication Date: (01/01/2018 TO 31/12/2024)]

K1 AND K2 AND K4

Title

[[Title: "animated character*"] OR [Title: "artificial agent*"] OR [Title: "artificial intelligence assistant*"] OR [Title: "assistive social agent*"] OR [Title: "communicative agent*"] OR [Title: "conversational agent*"] OR [Title: "companion agent"] OR [Title: "companion assistant*"] OR [Title: "conversational agent*"] OR [Title: "conversational assistant*"] OR [Title: "digital assistant*"] OR [Title: "embodied agent*"] OR [Title: "interactive agent*"] OR [Title: "interface agent*"] OR [Title: "pedagogical agent*"] OR [Title: "eca"] OR [Title: "relational agent*"] OR [Title: "relational assistant*"] OR [Title: "virtual agent*"] OR [Title: "virtual assistant*"] OR [Title: "virtual character"] OR [Title: "virtual coach*"] OR [Title: "virtual counselor*"] OR [Title: "virtual health counselor*"] OR [Title: "virtual health agent*"] OR [Title: "virtual health coach"] OR [Title: "virtual human"] OR [Title: "virtual therapist*"] OR [Title: "virtual advisor"] OR [Title: "artificial companion*"] OR [Title: "coaching system"] OR [Title: "chatbot"] OR [Title: "communication robot"] OR [Title: "virtual expert"] OR [Title: "virtual friend"] OR [Title: "virtual tutor"] OR [Title: "virtual instructor"] OR [Title: "virtual personal trainer"] OR [Title: "virtual companion*"] OR [Title: "virtual carer"] OR [Title: "virtual mentor"] OR [Title: "e-coach*"] OR [Title: "ecoach*"] OR [Title: "virtual coach"] OR [Title: "digital coach*"] OR [Title: "digital avatar"] OR [Title: "digital agent*"]] AND [[Title: "large language model*"] OR [Title: "llm*"] OR [Title: "bert"] OR [Title: "chatgpt"] OR [Title: "gpt*"] OR [Title: "llama"] OR [Title: "claude"] OR [Title: "gemini"] OR [Title: "bard"] OR [Title: "falcon"] OR [Title: "bart"] OR [Title: "palm"] OR [Title: "mistral"] OR [Title: "mixtral"] OR [Title: "bloom"] OR [Title: "phi"] OR [Title: "pretrained model"] OR [Title: "transformer*"] OR [Title: "generative ai"] OR [Title: "genai"]] AND [[Title: "executive function*"] OR [Title: "cognitive load"] OR [Title: "cognitive train*"] OR [Title: "cognitive therapy"] OR [Title: "cognitive intervention"] OR [Title: "memory train*"] OR [Title: "cognitive impair*"] OR [Title: "dementia"] OR [Title: "alzheimer"] OR [Title: "mci"] OR [Title: "cognitive decline"] OR [Title: "cognitive disorder*"] OR [Title: "cognitive deficit*"] OR [Title: "brain train*"] OR [Title: "brain workout"] OR [Title: "brain exercis*"] OR [Title: "cognit* retrain*"]] AND [E-Publication Date: (01/01/2018 TO 31/12/2024)]

Abstract

[[Abstract: "animated character*"] OR [Abstract: "artificial agent*"] OR [Abstract: "artificial intelligence assistant*"] OR [Abstract: "assistive social agent*"] OR [Abstract: "communicative agent*"] OR [Abstract: "conversational agent*"] OR [Abstract: "companion agent"] OR [Abstract: "companion assistant*"] OR [Abstract: "conversational agent*"] OR [Abstract: "conversational assistant*"] OR [Abstract: "digital assistant*"] OR [Abstract: "embodied agent*"] OR [Abstract: "interactive agent*"] OR [Abstract: "interface agent*"] OR [Abstract: "pedagogical agent*"] OR [Abstract: "eca"] OR [Abstract: "relational agent*"] OR [Abstract: "relational assistant*"] OR [Abstract: "virtual agent*"] OR [Abstract: "virtual assistant*"] OR [Abstract: "virtual character"] OR [Abstract: "virtual coach*"] OR [Abstract: "virtual counselor*"] OR [Abstract: "virtual health counselor*"] OR [Abstract: "virtual health agent*"] OR [Abstract: "virtual health coach"] OR [Abstract: "virtual human"] OR [Abstract: "virtual therapist*"] OR [Abstract: "virtual advisor"] OR [Abstract: "artificial companion*"] OR [Abstract: "coaching system"] OR [Abstract: "chatbot"] OR [Abstract: "communication robot"] OR [Abstract: "virtual expert"] OR [Abstract: "virtual friend"] OR [Abstract: "virtual tutor"] OR [Abstract: "virtual instructor"] OR [Abstract: "virtual personal trainer"] OR [Abstract: "virtual companion*"] OR [Abstract: "virtual carer"] OR [Abstract: "virtual mentor"] OR [Abstract: "e-coach*"] OR [Abstract: "ecoach*"] OR [Abstract: "virtual coach"] OR [Abstract: "digital coach*"] OR [Abstract: "digital avatar"] OR [Abstract: "digital agent*"]] AND [[Abstract: "large language model*"] OR [Abstract: "llm*"] OR [Abstract: "bert"] OR [Abstract: "chatgpt"] OR [Abstract: "gpt*"] OR [Abstract: "llama"] OR [Abstract: "claude"] OR [Abstract: "gemini"] OR [Abstract: "bard"] OR [Abstract: "falcon"] OR [Abstract: "bart"] OR [Abstract: "palm"] OR [Abstract: "mistral"] OR [Abstract: "mixtral"] OR [Abstract: "bloom"] OR [Abstract: "phi"] OR [Abstract: "pretrained model"] OR [Abstract: "transformer*"] OR [Abstract: "generative ai"] OR [Abstract: "genai"]] AND [[Abstract: "executive function*"] OR [Abstract: "cognitive load"] OR [Abstract: "cognitive train*"] OR [Abstract: "cognitive therapy"] OR [Abstract: "cognitive intervention"] OR [Abstract: "memory train*"] OR [Abstract: "cognitive impair*"] OR [Abstract: "dementia"] OR [Abstract: "alzheimer"] OR [Abstract: "mci"] OR [Abstract: "cognitive decline"] OR [Abstract: "cognitive disorder*"] OR [Abstract: "cognitive deficit*"] OR [Abstract: "brain train*"] OR [Abstract: "brain workout"] OR [Abstract: "brain exercis*"] OR [Abstract: "cognit* retrain*"]] AND [E-Publication Date: (01/01/2018 TO 31/12/2024)]

Keywords

[[Keywords: "animated character*"] OR [Keywords: "artificial agent*"] OR [Keywords: "artificial intelligence assistant*"] OR [Keywords: "assistive social agent*"] OR [Keywords: "communicative agent*"] OR [Keywords: "conversational agent*"] OR [Keywords: "companion agent"] OR [Keywords: "companion assistant*"] OR [Keywords: "conversational agent*"] OR [Keywords: "conversational assistant*"] OR [Keywords: "digital assistant*"] OR [Keywords: "embodied agent*"] OR [Keywords: "interactive agent*"] OR [Keywords: "interface agent*"] OR [Keywords: "pedagogical agent*"] OR [Keywords: "eca"] OR [Keywords: "relational agent*"] OR [Keywords: "relational assistant*"] OR [Keywords: "virtual agent*"] OR [Keywords: "virtual assistant*"] OR [Keywords: "virtual character"] OR [Keywords: "virtual coach*"] OR [Keywords: "virtual counselor*"] OR [Keywords: "virtual health counselor*"] OR [Keywords: "virtual health agent*"] OR [Keywords: "virtual health coach"] OR [Keywords: "virtual human"] OR [Keywords: "virtual therapist*"] OR [Keywords: "virtual advisor"] OR [Keywords: "artificial companion*"] OR [Keywords: "coaching system"] OR [Keywords: "chatbot"] OR [Keywords: "communication robot"] OR [Keywords: "virtual expert"] OR [Keywords: "virtual friend"] OR [Keywords: "virtual tutor"] OR [Keywords: "virtual instructor"] OR [Keywords: "virtual personal trainer"] OR [Keywords: "virtual companion*"] OR [Keywords: "virtual carer"] OR [Keywords: "virtual mentor"] OR [Keywords: "e-coach*"] OR [Keywords: "ecoach*"] OR [Keywords: "virtual coach"] OR [Keywords: "digital coach*"] OR [Keywords: "digital avatar"] OR [Keywords: "digital agent*"]] AND [[Keywords: "large language model*"] OR [Keywords: "llm*"] OR [Keywords: "bert"] OR [Keywords: "chatgpt"] OR [Keywords: "gpt*"] OR [Keywords: "llama"] OR [Keywords: "claude"] OR [Keywords: "gemini"] OR [Keywords: "bard"] OR [Keywords: "falcon"] OR [Keywords: "bart"] OR [Keywords: "palm"] OR [Keywords: "mistral"] OR [Keywords: "mixtral"] OR [Keywords: "bloom"] OR [Keywords: "phi"] OR [Keywords: "pretrained model"] OR [Keywords: "transformer*"] OR [Keywords: "generative ai"] OR [Keywords: "genai"]] AND [[Keywords: "executive function*"] OR [Keywords: "cognitive load"] OR [Keywords: "cognitive train*"] OR [Keywords: "cognitive therapy"] OR [Keywords: "cognitive intervention"] OR [Keywords: "memory train*"] OR [Keywords: "cognitive impair*"] OR [Keywords: "dementia"] OR [Keywords: "alzheimer"] OR [Keywords: "mci"] OR [Keywords: "cognitive decline"] OR [Keywords: "cognitive disorder*"] OR [Keywords: "cognitive deficit*"] OR [Keywords: "brain train*"] OR [Keywords: "brain workout"] OR [Keywords: "brain exercis*"] OR [Keywords: "cognit* retrain*"]] AND [E-Publication Date: (01/01/2018 TO 31/12/2024)]

Pubmed

K1 AND K2 AND K3

Title & Abstract

(("animated character*"[Title/Abstract] OR "artificial agent*"[Title/Abstract] OR "artificial intelligence assistant*"[Title/Abstract] OR "assistive social agent*"[Title/Abstract] OR "communicative agent*"[Title/Abstract] OR "conversational agent*"[Title/Abstract] OR "companion agent"[Title/Abstract] OR "companion assistant*"[Title/Abstract] OR "conversational agent*"[Title/Abstract] OR "conversational assistant*"[Title/Abstract] OR "digital assistant*"[Title/Abstract] OR "embodied agent*"[Title/Abstract] OR "interactive agent*"[Title/Abstract] OR "interface agent*"[Title/Abstract] OR "pedagogical agent*"[Title/Abstract] OR "ECA"[Title/Abstract] OR "relational agent*"[Title/Abstract] OR "relational assistant*"[Title/Abstract] OR "virtual agent*"[Title/Abstract] OR "virtual assistant*"[Title/Abstract] OR "virtual character"[Title/Abstract] OR "virtual coach*"[Title/Abstract] OR "virtual counselor*"[Title/Abstract] OR "virtual health counselor*"[Title/Abstract] OR "virtual health agent*"[Title/Abstract] OR "virtual health coach"[Title/Abstract] OR "virtual human"[Title/Abstract] OR "virtual therapist*"[Title/Abstract] OR "virtual advisor"[Title/Abstract] OR "artificial companion*"[Title/Abstract] OR "coaching system"[Title/Abstract] OR "chatbot"[Title/Abstract] OR "communication robot"[Title/Abstract] OR "virtual expert"[Title/Abstract] OR "virtual friend"[Title/Abstract] OR "virtual tutor"[Title/Abstract] OR "virtual instructor"[Title/Abstract] OR "virtual personal trainer"[Title/Abstract] OR "virtual companion*"[Title/Abstract] OR "virtual carer"[Title/Abstract] OR "virtual mentor"[Title/Abstract] OR "e-coach*"[Title/Abstract] OR "ecoach*"[Title/Abstract] OR "virtual coach"[Title/Abstract] OR "digital coach*"[Title/Abstract] OR "digital avatar"[Title/Abstract] OR "digital agent*"[Title/Abstract]) AND ("Large Language Model*"[Title/Abstract] OR "LLM*"[Title/Abstract] OR "BERT"[Title/Abstract] OR "ChatGPT"[Title/Abstract] OR "GPT*"[Title/Abstract] OR "Llama"[Title/Abstract] OR "Claude"[Title/Abstract] OR "Gemini"[Title/Abstract] OR "Bard"[Title/Abstract] OR "Falcon"[Title/Abstract] OR "BART"[Title/Abstract] OR "PaLM"[Title/Abstract] OR "Mistral"[Title/Abstract] OR "Mixtral"[Title/Abstract] OR "Bloom"[Title/Abstract] OR "Phi"[Title/Abstract] OR "pretrained model"[Title/Abstract] OR "transformer*"[Title/Abstract] OR "generative AI"[Title/Abstract] OR "genAI"[Title/Abstract])) AND ("physical activity"[Title/Abstract] OR "physical* activ*"[Title/Abstract] OR "sport*"[Title/Abstract] OR "physical exercis*"[Title/Abstract] OR "workout"[Title/Abstract] OR "exergam*"[Title/Abstract] OR "fitness gam*"[Title/Abstract] OR "digital play"[Title/Abstract] OR "physical action gam*"[Title/Abstract] OR "health gam*"[Title/Abstract] OR "physical fitness"[Title/Abstract] OR "physical education"[Title/Abstract] OR "motor

activity"[Title/Abstract] OR "walking"[Title/Abstract] OR "running"[Title/Abstract] OR "cycling"[Title/Abstract] OR "swimming"[Title/Abstract] OR "hiking"[Title/Abstract] OR "leisure activity"[Title/Abstract])

K1 AND K2 AND K4

Title & Abstract

(("animated character*"[Title/Abstract] OR "artificial agent*"[Title/Abstract] OR "artificial intelligence assistant*"[Title/Abstract] OR "assistive social agent*"[Title/Abstract] OR "communicative agent*"[Title/Abstract] OR "conversational agent*"[Title/Abstract] OR "companion agent"[Title/Abstract] OR "companion assistant*"[Title/Abstract] OR "conversational agent*"[Title/Abstract] OR "conversational assistant*"[Title/Abstract] OR "digital assistant*"[Title/Abstract] OR "embodied agent*"[Title/Abstract] OR "interactive agent*"[Title/Abstract] OR "interface agent*"[Title/Abstract] OR "pedagogical agent*"[Title/Abstract] OR "ECA"[Title/Abstract] OR "relational agent*"[Title/Abstract] OR "relational assistant*"[Title/Abstract] OR "virtual agent*"[Title/Abstract] OR "virtual assistant*"[Title/Abstract] OR "virtual character"[Title/Abstract] OR "virtual coach*"[Title/Abstract] OR "virtual counselor*"[Title/Abstract] OR "virtual health counselor*"[Title/Abstract] OR "virtual health agent*"[Title/Abstract] OR "virtual health coach"[Title/Abstract] OR "virtual human"[Title/Abstract] OR "virtual therapist*"[Title/Abstract] OR "virtual advisor"[Title/Abstract] OR "artificial companion*"[Title/Abstract] OR "coaching system"[Title/Abstract] OR "chatbot"[Title/Abstract] OR "communication robot"[Title/Abstract] OR "virtual expert"[Title/Abstract] OR "virtual friend"[Title/Abstract] OR "virtual tutor"[Title/Abstract] OR "virtual instructor"[Title/Abstract] OR "virtual personal trainer"[Title/Abstract] OR "virtual companion*"[Title/Abstract] OR "virtual carer"[Title/Abstract] OR "virtual mentor"[Title/Abstract] OR "e-coach*"[Title/Abstract] OR "ecoach*"[Title/Abstract] OR "virtual coach"[Title/Abstract] OR "digital coach*"[Title/Abstract] OR "digital avatar"[Title/Abstract] OR "digital agent*"[Title/Abstract]) AND ("Large Language Model*"[Title/Abstract] OR "LLM*"[Title/Abstract] OR "BERT"[Title/Abstract] OR "ChatGPT"[Title/Abstract] OR "GPT*"[Title/Abstract] OR "Llama"[Title/Abstract] OR "Claude"[Title/Abstract] OR "Gemini"[Title/Abstract] OR "Bard"[Title/Abstract] OR "Falcon"[Title/Abstract] OR "BART"[Title/Abstract] OR "PaLM"[Title/Abstract] OR "Mistral"[Title/Abstract] OR "Mixtral"[Title/Abstract] OR "Bloom"[Title/Abstract] OR "Phi"[Title/Abstract] OR "pretrained model"[Title/Abstract] OR "transformer*"[Title/Abstract] OR "generative AI"[Title/Abstract] OR "genAI"[Title/Abstract])) AND ("executive function*"[Title/Abstract] OR "cognitive load"[Title/Abstract] OR "cognitive train*"[Title/Abstract] OR "cognitive therapy"[Title/Abstract] OR "cognitive intervention"[Title/Abstract] OR "memory train*"[Title/Abstract] OR "cognitive impair*"[Title/Abstract] OR "dementia"[Title/Abstract] OR "Alzheimer"[Title/Abstract] OR "MCI"[Title/Abstract] OR "cognitive decline"[Title/Abstract] OR "cognitive disorder*"[Title/Abstract] OR "cognitive deficit*"[Title/Abstract] OR "brain train*"[Title/Abstract] OR "brain workout"[Title/Abstract] OR "brain exercis*"[Title/Abstract] OR "cognit* retrain*"[Title/Abstract])
