# Supplementary material for: Large Language Model–Based Agents for Physical Activity and Cognitive Training: Scoping Review
Source: JMIR AI. 2026 Mar 12;5:e80123. doi: 10.2196/80123 (PMC12981376; doi:10.2196/80123)
Supplement: Multimedia Appendix 1 [file ai-v5-e80123-s001.zip › supplementary_materials_large_language_models_pa_ct_scoping_review/02_queries/022_queries_keywords_table.pdf]

Supplementary Material - Large Language Model-Based Agents for Physical Activity and Cognitive Training: A Scoping Review: Queries' keywords

Introduction

This document presents the complete list of keyword groups and corresponding database queries used in the review Large Language Model-Based Agents for Physical Activity and Cognitive Training: A Scoping Review. The queries were executed across major academic databases as part of the study identification process. Detailed descriptions of how the keyword groups were structured and combined in the search strategy are provided in the main article and in the accompanying FAIR repository.

Keywords Table

Keywords Group ID	List of keywords	Query
K1<br>Agents	"animated character*"<br>"artificial agent*"<br>"artificial intelligence assistant*"<br>"assistive social agent*"<br>"communicative agent*"<br>“conversational agent*”<br>"companion agent"<br>"companion assistant*"<br>"conversational agent*"<br>"conversational assistant*"<br>"digital assistant*"<br>"embodied agent*"<br>"interactive agent*"<br>"interface agent*"<br>"pedagogical agent*"<br>"ECA"<br>"relational agent*"<br>"relational assistant*"<br>"virtual agent*"<br>"virtual assistant*"<br>"virtual character"<br>"virtual coach*"<br>"virtual counselor*"<br>"virtual health counselor*"<br>"virtual health agent*"<br>"virtual health coach"<br>"virtual human"	"animated character*" OR "artificial agent*" OR "artificial intelligence assistant*" OR "assistive social agent*" OR "communicative agent*" OR “conversational agent*” OR "companion agent" OR "companion assistant*" OR "conversational agent*" OR "conversational assistant*" OR "digital assistant*" OR "embodied agent*" OR "interactive agent*" OR "interface agent*" OR "pedagogical agent*" OR "ECA" OR "relational agent*" OR "relational assistant*" OR "virtual agent*" OR "virtual assistant*" OR "virtual character" OR "virtual coach*" OR "virtual counselor*" OR "virtual health counselor*" OR "virtual health agent*" OR "virtual health coach"

	"virtual therapist*"<br>"virtual advisor"<br>"artificial companion*"<br>"coaching system"<br>"chatbot"<br>"communication robot"<br>"virtual expert"<br>"virtual friend"<br>"virtual tutor"<br>"virtual instructor"<br>"virtual personal trainer"<br>"virtual companion*"<br>"virtual carer"<br>"virtual mentor"<br>"e-coach*"<br>"ecoach*"<br>"virtual coach"<br>"digital coach*"<br>"digital avatar"<br>"digital agent*"	OR "virtual human" OR "virtual therapist*" OR "virtual advisor" OR "artificial companion*" OR "coaching system" OR "chatbot" OR "communication robot" OR "virtual expert" OR "virtual friend" OR "virtual tutor" OR "virtual instructor" OR "virtual personal trainer" OR "virtual companion*" OR "virtual carer" OR "virtual mentor" OR "e-coach*" OR "ecoach*" OR "virtual coach" OR "digital coach*" OR "digital avatar" OR "digital agent*"
K2<br>Technology	“Large Language Model*”<br>“LLM*”<br>“BERT”<br>“ChatGPT”<br>“GPT*”<br>“Llama”<br>“Claude”<br>“Gemini”<br>“Bard”<br>“Falcon”<br>“BART”<br>“PaLM”<br>“Mistral”<br>“Mixtral”<br>“Bloom”<br>“Phi”	[illegible]R "ChatGPT" OR "GPT*" OR [illegible]alcon" OR "BART" OR "PaLM"

K3<br>Physical Activity	“physical activity"<br>“physically active”<br>"sport*"<br>"physical exercis*"<br>“physical training”<br>“workout”<br>“exergam*”<br>“fitness gam*”<br>“digital play”<br>“physical action gam*”<br>“health gam*”<br>“physical fitness”<br>“physical education”<br>“motor activity”<br>“walking”<br>“running”<br>“cycling”<br>“swimming”<br>“hiking”<br>“leisure activity”	"physical activity" OR "physical* activ*" OR "sport*" OR "physical exercis*" OR "workout" OR “exergam*” OR “fitness gam*” OR "digital play" OR "physical action gam*" OR "health gam*" OR "physical fitness" OR "physical education" OR "motor activity" OR "walking" OR "running" OR "cycling" OR "swimming" OR "hiking" OR "leisure activity"
K4<br>Cognition	“executive function*”<br>“cognitive load”<br>“cognitive train*”<br>“cognitive therapy”<br>“cognitive intervention”<br>“memory train*”<br>“cognitive impair*”<br>“dementia”<br>“Alzheimer”<br>“MCI”<br>“cognitive decline”<br>”cognitive disorder*”<br>“cognitive deficit*”<br>“brain train*”<br>“brain workout”<br>“brain exercis*”<br>"cognit* retrain*”	"executive function*" OR "cognitive load" OR "cognitive train*" OR "cognitive therapy" OR "cognitive intervention" OR "memory train*" OR "cognitive impair*" OR "dementia" OR "Alzheimer" OR "MCI" OR "cognitive decline" OR "cognitive disorder*" OR "cognitive deficit*" OR "brain train*" OR "brain workout" OR "brain exercis*" OR "cognit* retrain*"
