# Supplementary material for: Large Language Model–Based Agents for Physical Activity and Cognitive Training: Scoping Review
Source: JMIR AI. 2026 Mar 12;5:e80123. doi: 10.2196/80123 (PMC12981376; doi:10.2196/80123)
Supplement: Multimedia Appendix 1 [file ai-v5-e80123-s001.zip › supplementary_materials_large_language_models_pa_ct_scoping_review/05_data_analysis/052_quantitative_analysis/0521_data_analysis_report.html]

Supplementary Material: Quantitative Data Analysis Notebook for Scoping Review


# Supplementary Material: Quantitative Data Analysis Notebook for Scoping Review

#### 2025-07-02

```
## ── Attaching core tidyverse packages ──────────────────────── tidyverse 2.0.0 ──
## ✔ dplyr     1.1.4     ✔ readr     2.1.5
## ✔ forcats   1.0.0     ✔ stringr   1.5.1
## ✔ ggplot2   3.5.1     ✔ tibble    3.2.1
## ✔ lubridate 1.9.4     ✔ tidyr     1.3.1
## ✔ purrr     1.0.4     
## ── Conflicts ────────────────────────────────────────── tidyverse_conflicts() ──
## ✖ dplyr::filter() masks stats::filter()
## ✖ dplyr::lag()    masks stats::lag()
## ℹ Use the conflicted package (<http://conflicted.r-lib.org/>) to force all conflicts to become errors
## Loading required package: viridisLite
```

# Introduction

This document provides the R Markdown notebook used to conduct the
quantitative data analysis and generate the descriptive statistics
charts presented in the main manuscript of the scoping review, “Large
Language Model-Based Agents for Physical Activity and Cognitive
Training: A Scoping Review.”

# Contents

This R Markdown notebook includes:

- **Data Loading and Preparation:** Steps for loading
  the `042_consensus_extracted_dataset.xlsx` dataset and
  initial data manipulation, including the creation of flag variables for
  targeted domains.
- **Descriptive Statistics and Visualization Code:** R
  code for generating various bar charts that illustrate key
  characteristics of the included studies, such as:

  - Distribution of publication types.
  - Prevalence of targeted domains (physical activity vs. cognitive
    training).
  - Specific LLM models identified in the reviewed
    literature.
  - Types and designs of the included studies.
  - Social roles attributed to conversational agents.
  - Interaction modalities and dialogue initiatives.
  - Deployment platforms utilized.
- **Generated Charts:** The output HTML file displays
  all the charts directly, demonstrating the results of the R
  code.

The underlying
dataset, `042_consensus_extracted_dataset.xlsx`, along with
the rest of the supplementary materials, is available in the FAIR
repository mentioned in the main manuscript.

# Loading the dataset

```
df <- read_excel("042_consensus_extracted_dataset.xlsx")
```

```
## New names:
## • `Title` -> `Title...3`
## • `Title` -> `Title...5`
```

## Data preparation

```
df <- df %>%
  mutate(Targeted_domain_PA_Flag = if_else(str_detect(`Targeted domain`, regex("Physical activity", ignore_case = TRUE)), 1, 0)) %>%
  mutate(Targeted_domain_CT_Flag = if_else(str_detect(`Targeted domain`, regex("Cognitive", ignore_case = TRUE)), 1, 0)) %>%
  mutate()
```

# Descriptive statistics charts

## Publication types

```
ggplot(df, aes(x = `Publication type`, fill = `Publication type`)) +
  geom_bar() +
  labs(title = "Publication types", x = "Publication type", y = "Count") +
  scale_fill_viridis(discrete = TRUE, option = "D") +
  guides(fill="none")
```

## Target domains

```
df_l <- df %>%
  select(Targeted_domain_PA_Flag, Targeted_domain_CT_Flag) %>%
  pivot_longer(cols = everything(), names_to = "Targeted_domain", values_to = "Value") %>%
  mutate(Targeted_domain = case_when(
    Targeted_domain == "Targeted_domain_PA_Flag" ~ "Physical Activity",
    Targeted_domain == "Targeted_domain_CT_Flag" ~ "Cognitive Training"
  ))

ggplot(df_l %>% filter(Value == 1), aes(x = Targeted_domain, fill = Targeted_domain)) +
  geom_bar() +
  labs(title = "Target domains", x = "Target domains", y = "Count") +
  scale_fill_viridis(discrete = TRUE, option = "D") +
  guides(fill="none")
```

## Models used

```
df_models <- df %>%
  separate_rows(`Model Sanitized`, sep=",") %>%
  mutate(`Model Sanitized` = str_trim(`Model Sanitized`)) %>%
  group_by(`Model Sanitized`) %>%
  summarise(Count = n()) %>%
  arrange(desc(Count))

ggplot(df_models, aes(x = fct_reorder(`Model Sanitized`, Count), y = Count, fill = `Model Sanitized`)) +
  geom_col() +
  labs(title = "Model used", x = "Model names", y = "Count") +
  scale_fill_viridis(discrete = TRUE, option = "D") +
  theme(axis.text.x = element_text(angle = 45, hjust = 1)) +
  guides(fill="none")
```

## Study types

```
df_study_design <- df %>%
  mutate(Study_design_sanitized = gsub("^Other: ", "", `Study design`)) %>%
  group_by(Study_design_sanitized) %>%
  summarise(Count = n()) %>%
  arrange(desc(Count))

df_study_type <- df %>%
  mutate(Study_type_sanitized = gsub("^Other: ", "", `Study type`)) %>%
  separate_rows(Study_type_sanitized, sep=",") %>%
  group_by(Study_type_sanitized) %>%
  summarise(Count = n()) %>%
  arrange(desc(Count))

ggplot(df_study_type, aes(x = fct_reorder(Study_type_sanitized, Count), y = Count, fill = Study_type_sanitized)) +
  geom_col() +
  labs(title = "Study types in the reviewed dataset", x = "Study design", y = "Count") +
  scale_fill_viridis(discrete = TRUE, option = "D") +
  theme(axis.text.x = element_text(angle = 45, hjust = 1)) +
  guides(fill="none")
```

## Roles

```
df_roles <- df %>%
  separate_rows(Role, sep=",") %>%
  group_by(Role) %>%
  summarise(Count = n()) %>%
  arrange(desc(Count))


ggplot(df_roles, aes(x = fct_reorder(Role, Count), y = Count, fill = Role)) +
  geom_col() +
  labs(title = "Conversational Agents' Roles in reviewed dataset", x = "Role", y = "Count") +
  scale_fill_viridis(discrete = TRUE, option = "D") +
  theme(axis.text.x = element_text(angle = 45, hjust = 1)) +
  guides(fill="none")
```

## Interaction modality

```
df_interaction <- df %>%
  separate_rows(`Interaction modality`, sep=",") %>%
  mutate(`Interaction modality` = str_trim(`Interaction modality`)) %>%
  group_by(`Interaction modality`) %>%
  summarise(Count = n()) %>%
  arrange(desc(Count))


ggplot(df_interaction, aes(x = fct_reorder(`Interaction modality`, Count), y = Count, fill = `Interaction modality`)) +
  geom_col() +
  labs(title = "Conversational Agents' Roles in reviewed dataset", x = "Interaction modality", y = "Count") +
  scale_fill_viridis(discrete = TRUE, option = "D") +
  theme(axis.text.x = element_text(angle = 45, hjust = 1)) +
  guides(fill="none")
```

## Dialogue initiative

```
df_dialog <- df %>%
  separate_rows(`Dialogue initiative`, sep=",") %>%
  mutate(`Dialogue initiative` = str_trim(`Dialogue initiative`)) %>%
  group_by(`Dialogue initiative`) %>%
  summarise(Count = n()) %>%
  arrange(desc(Count))


ggplot(df_dialog, aes(x = fct_reorder(`Dialogue initiative`, Count), y = Count, fill = `Dialogue initiative`)) +
  geom_col() +
  labs(title = "Dialogue initiators in reviewed dataset", x = "Dialogue Initiator", y = "Count") +
  scale_fill_viridis(discrete = TRUE, option = "D") +
  theme(axis.text.x = element_text(angle = 45, hjust = 1)) +
  guides(fill="none")
```

## Deployment platform

```
df_platforms <- df %>%
  separate_rows(`Deployment platform`, sep=",") %>%
  mutate(`Deployment platform` = str_trim(`Deployment platform`)) %>%
  group_by(`Deployment platform`) %>%
  summarise(Count = n()) %>%
  arrange(desc(Count))


ggplot(df_platforms, aes(x = fct_reorder(`Deployment platform`, Count), y = Count, fill = `Deployment platform`)) +
  geom_col() +
  labs(title = "Deployment platform used in reviewed dataset", x = "Deployment platforms", y = "Count") +
  scale_fill_viridis(discrete = TRUE, option = "D") +
  theme(axis.text.x = element_text(angle = 45, hjust = 1)) +
  guides(fill="none")
```
