# Supplementary figures and images for: Large Language Model–Based Agents for Physical Activity and Cognitive Training: Scoping Review
Source: JMIR AI. 2026 Mar 12;5:e80123. doi: 10.2196/80123 (PMC12981376; doi:10.2196/80123)

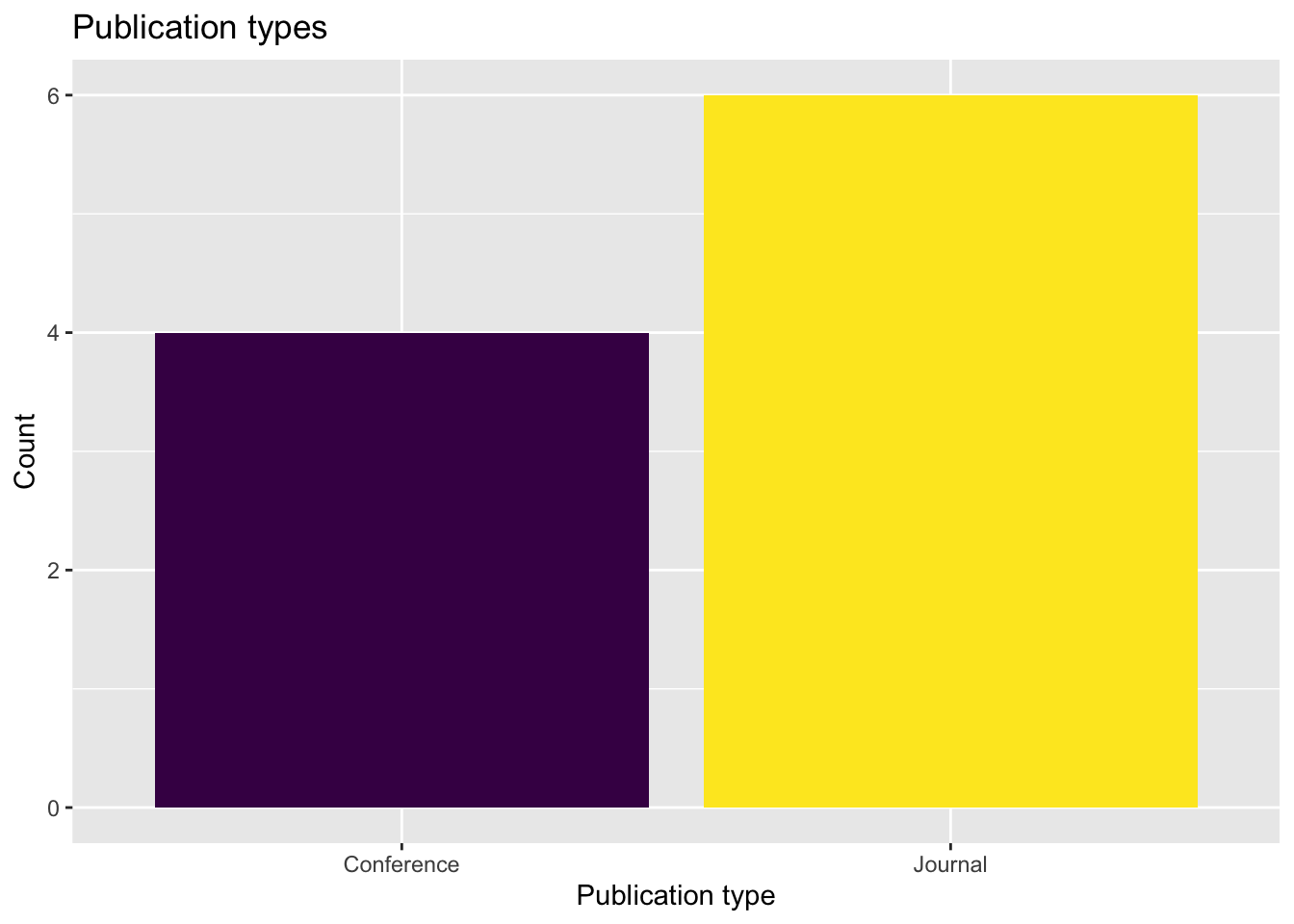

Supplement: Multimedia Appendix 1 [file ai-v5-e80123-s001.zip › supplementary_materials_large_language_models_pa_ct_scoping_review/05_data_analysis/052_quantitative_analysis/0523_data_analysis_figures/bar-publication-types-1.png]

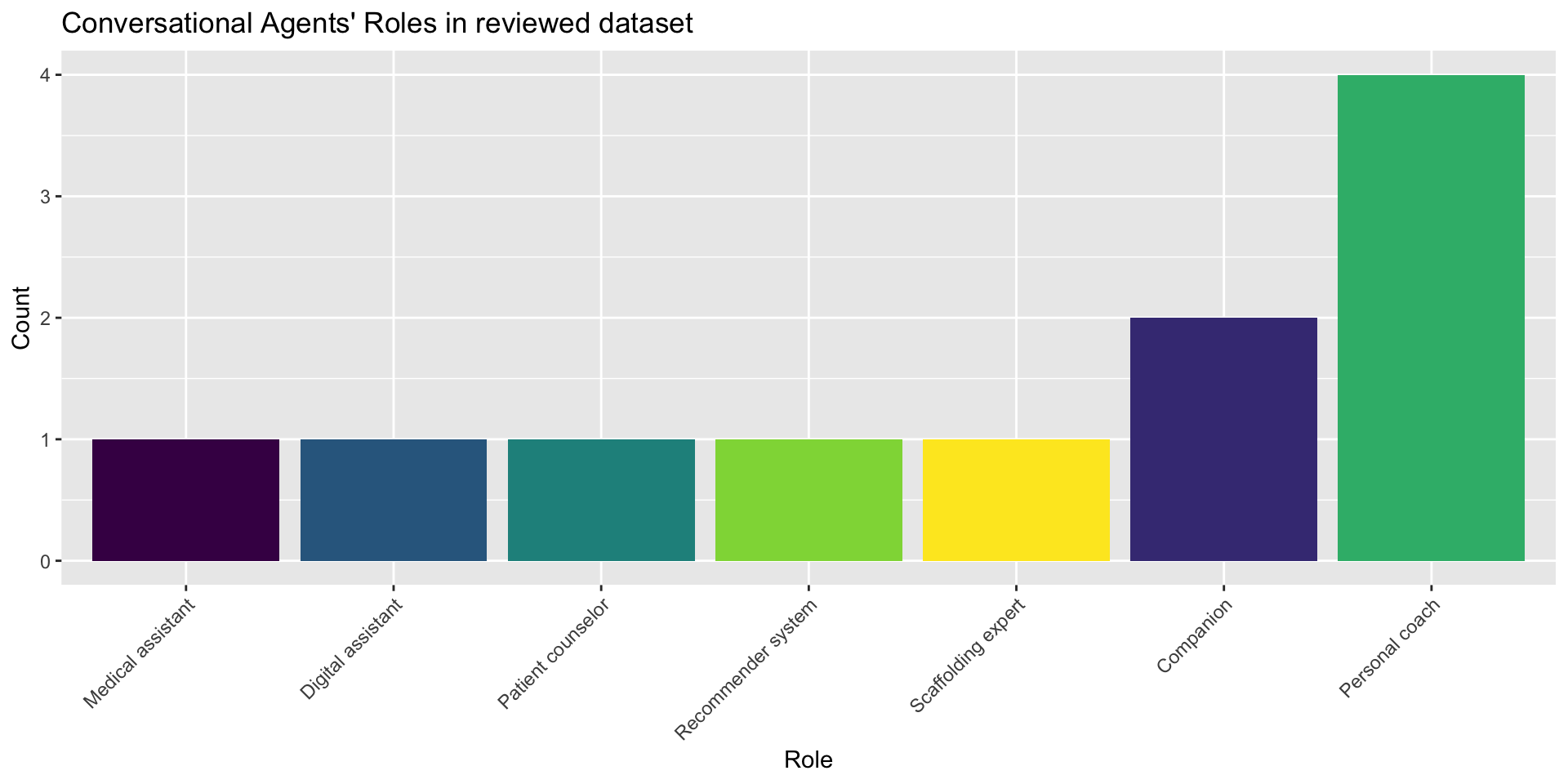

Supplement: Multimedia Appendix 1 [file ai-v5-e80123-s001.zip › supplementary_materials_large_language_models_pa_ct_scoping_review/05_data_analysis/052_quantitative_analysis/0523_data_analysis_figures/bar-ca-roles-1.png]

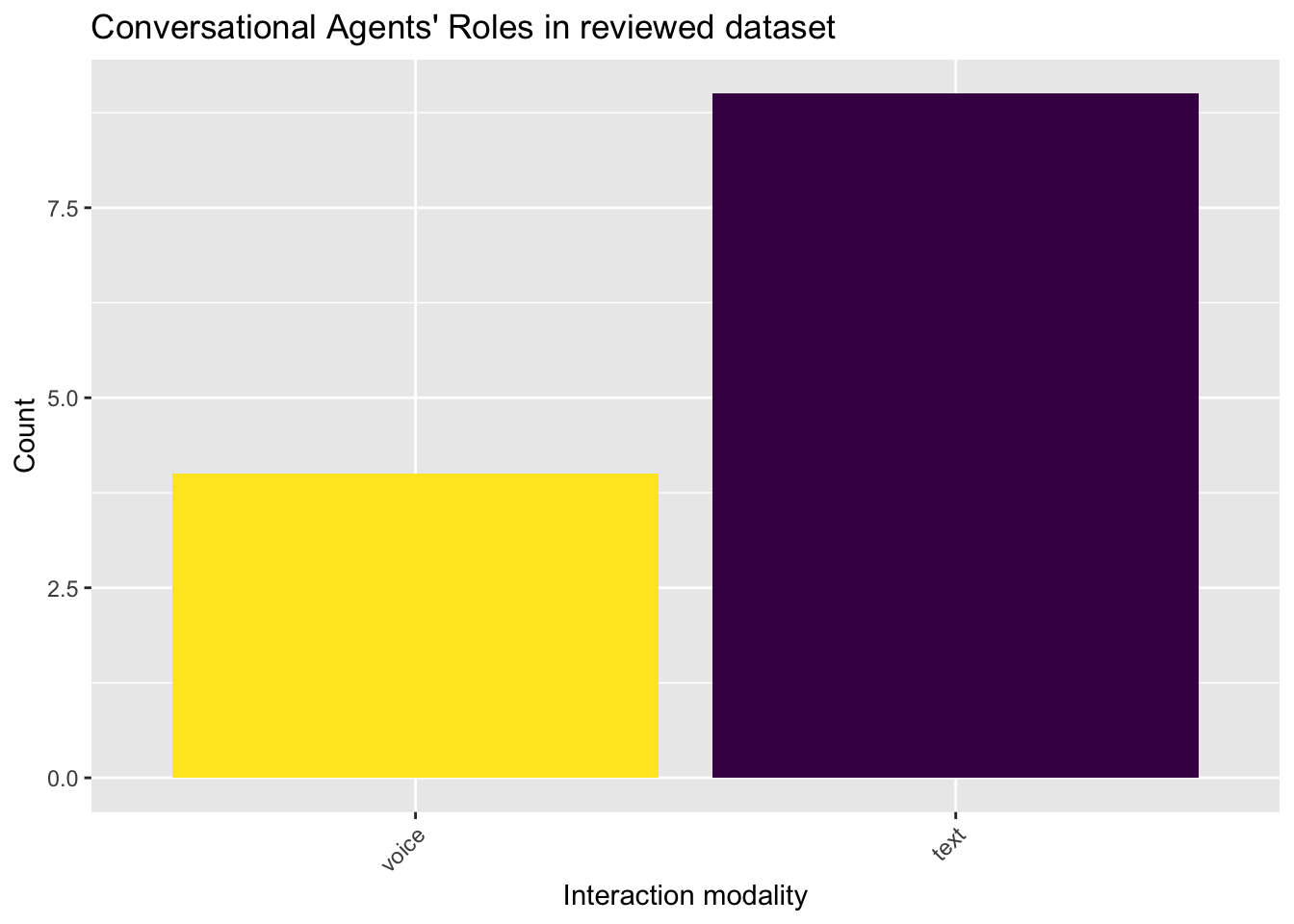

Supplement: Multimedia Appendix 1 [file ai-v5-e80123-s001.zip › supplementary_materials_large_language_models_pa_ct_scoping_review/05_data_analysis/052_quantitative_analysis/0523_data_analysis_figures/bar-interaction-modalities-1.png]

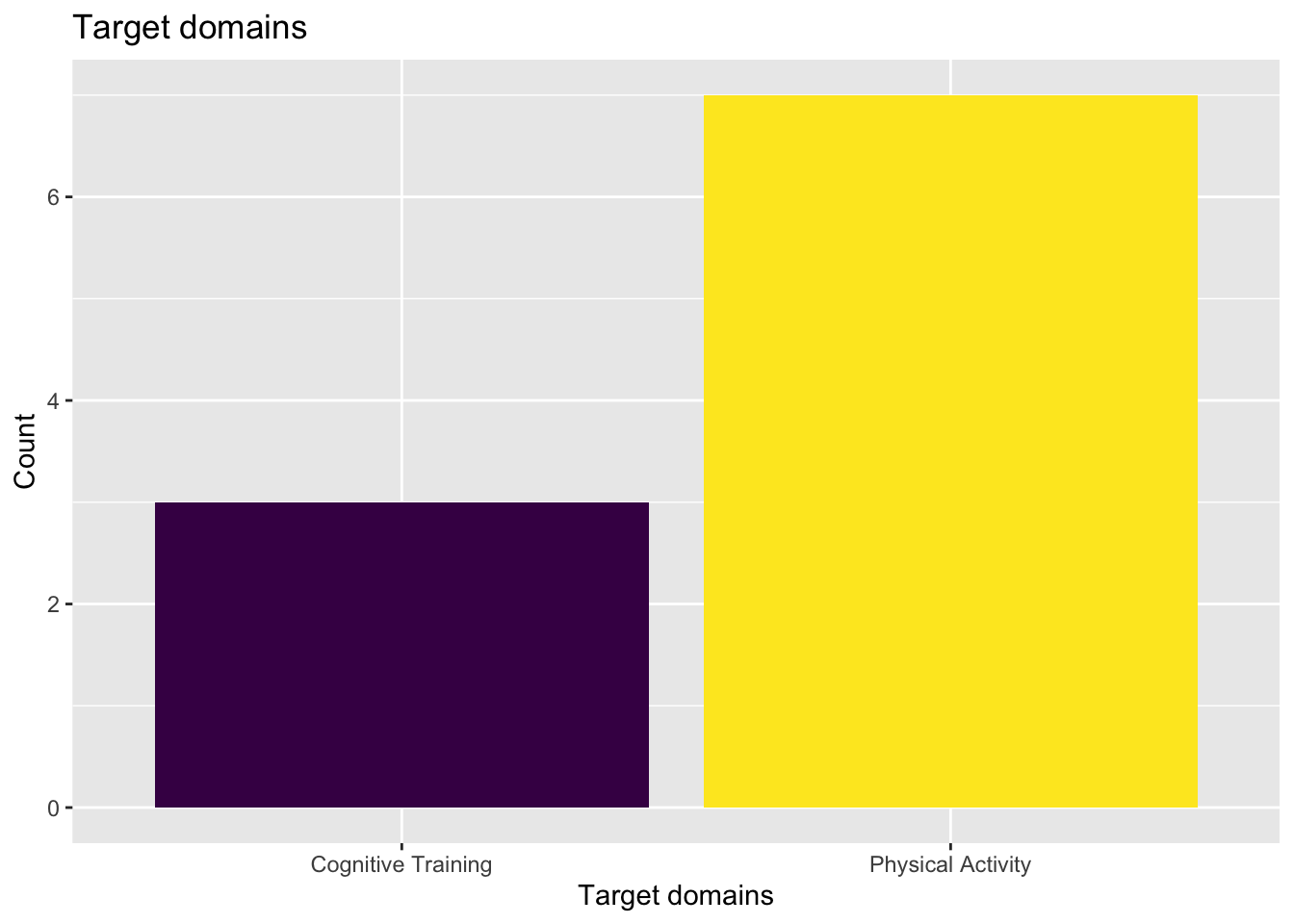

Supplement: Multimedia Appendix 1 [file ai-v5-e80123-s001.zip › supplementary_materials_large_language_models_pa_ct_scoping_review/05_data_analysis/052_quantitative_analysis/0523_data_analysis_figures/bar-target-domains-1.png]

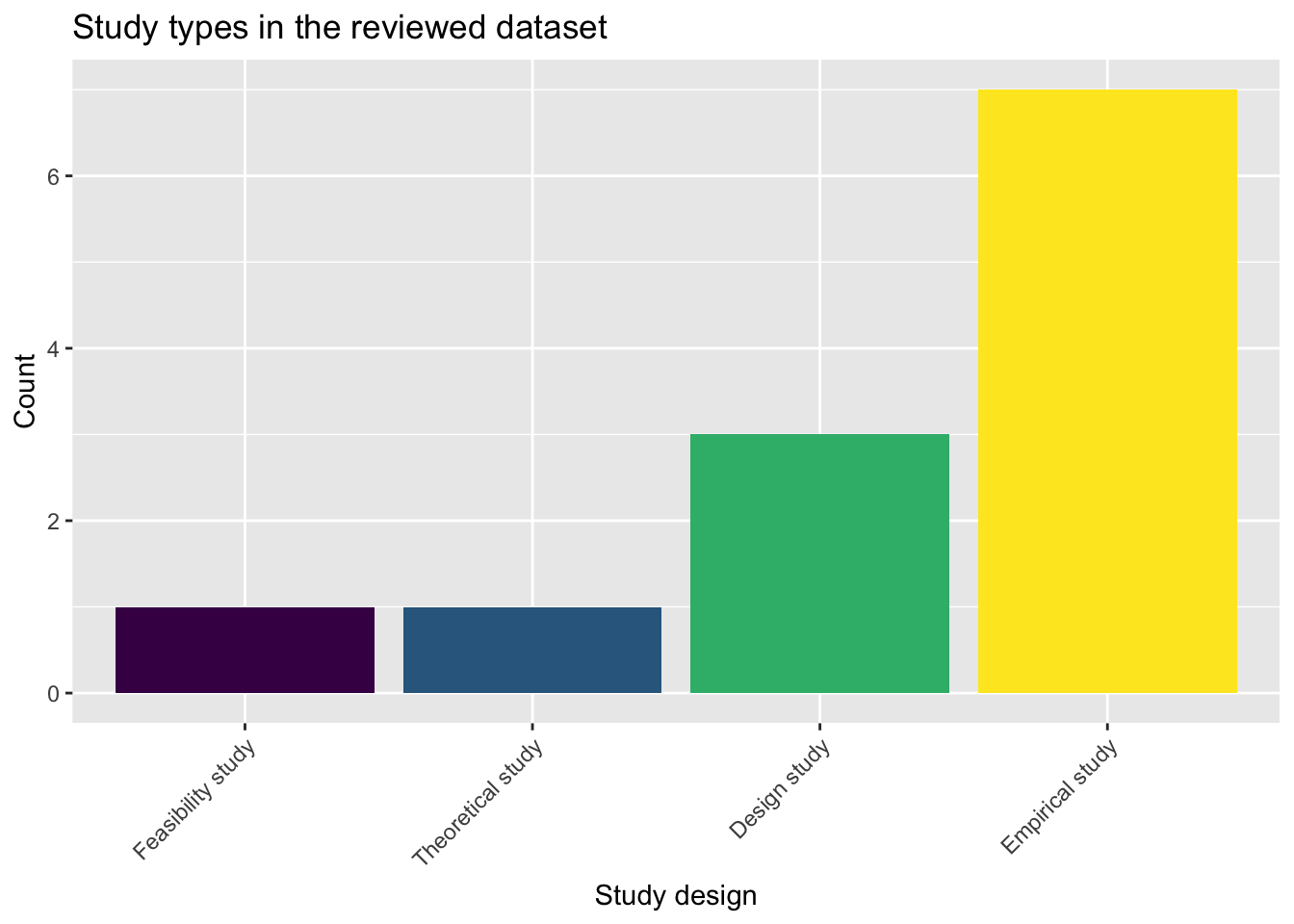

Supplement: Multimedia Appendix 1 [file ai-v5-e80123-s001.zip › supplementary_materials_large_language_models_pa_ct_scoping_review/05_data_analysis/052_quantitative_analysis/0523_data_analysis_figures/bar-study-types-1.png]

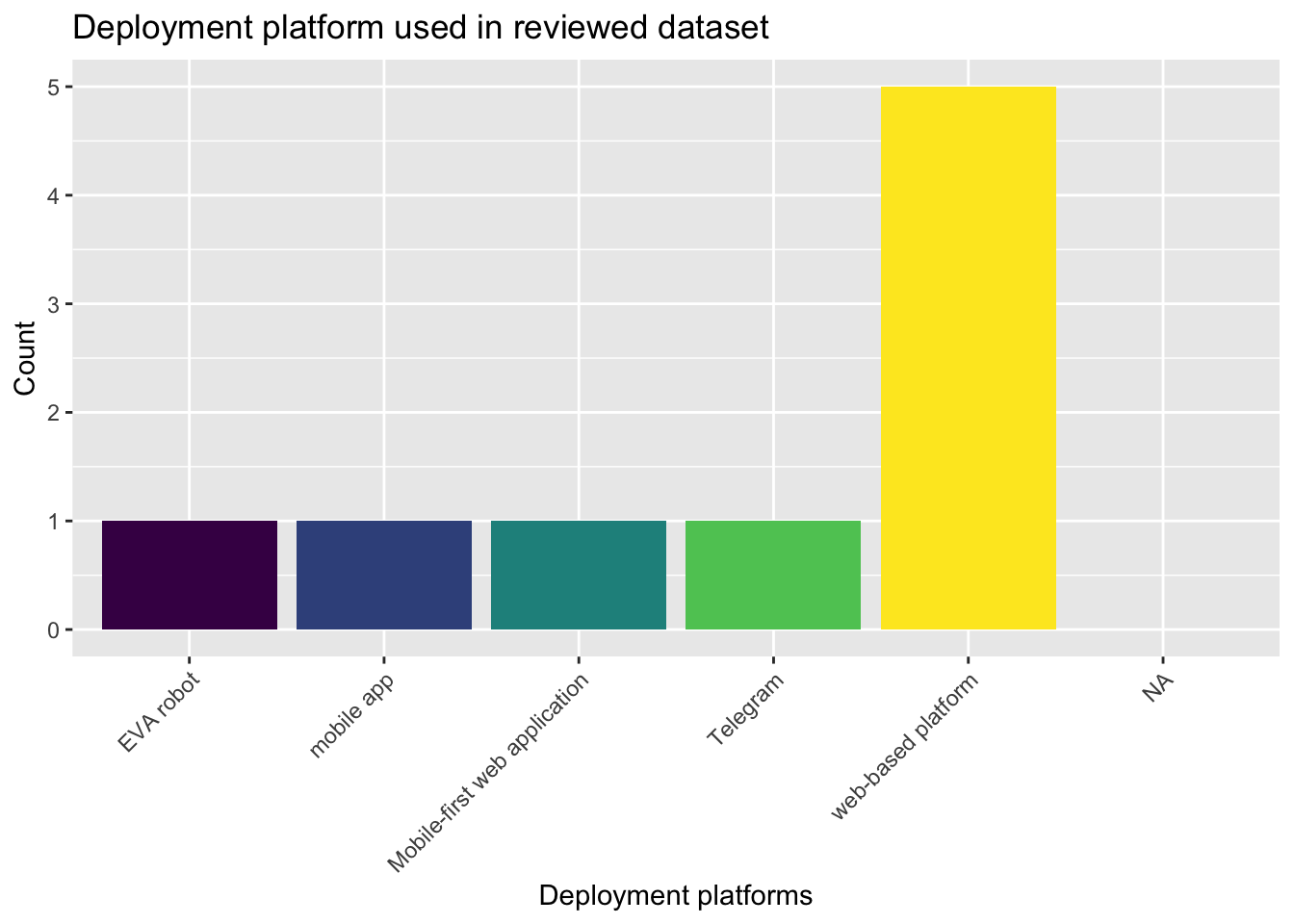

Supplement: Multimedia Appendix 1 [file ai-v5-e80123-s001.zip › supplementary_materials_large_language_models_pa_ct_scoping_review/05_data_analysis/052_quantitative_analysis/0523_data_analysis_figures/bar-deployment-platforms-1.png]

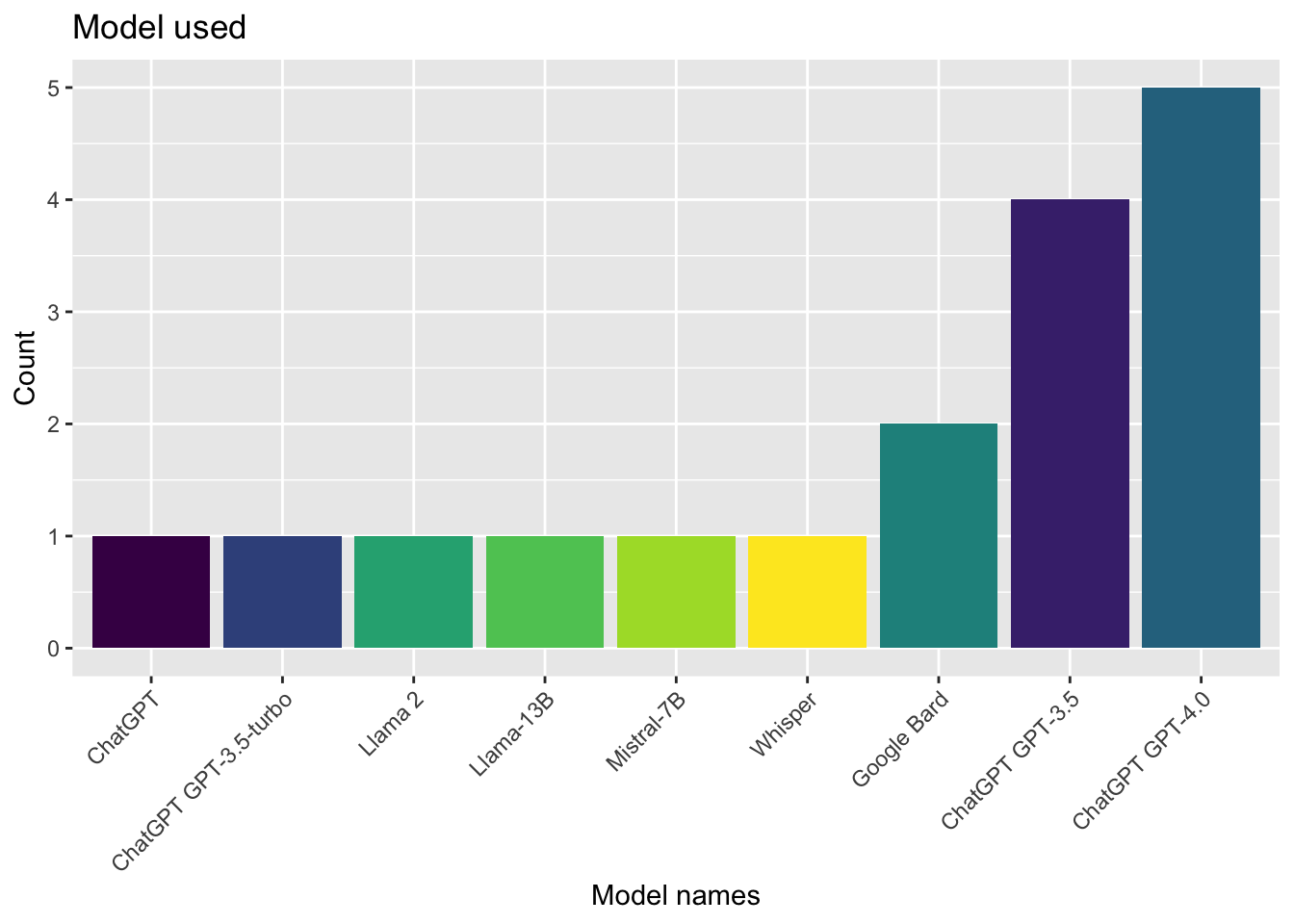

Supplement: Multimedia Appendix 1 [file ai-v5-e80123-s001.zip › supplementary_materials_large_language_models_pa_ct_scoping_review/05_data_analysis/052_quantitative_analysis/0523_data_analysis_figures/bar-model-used-1.png]

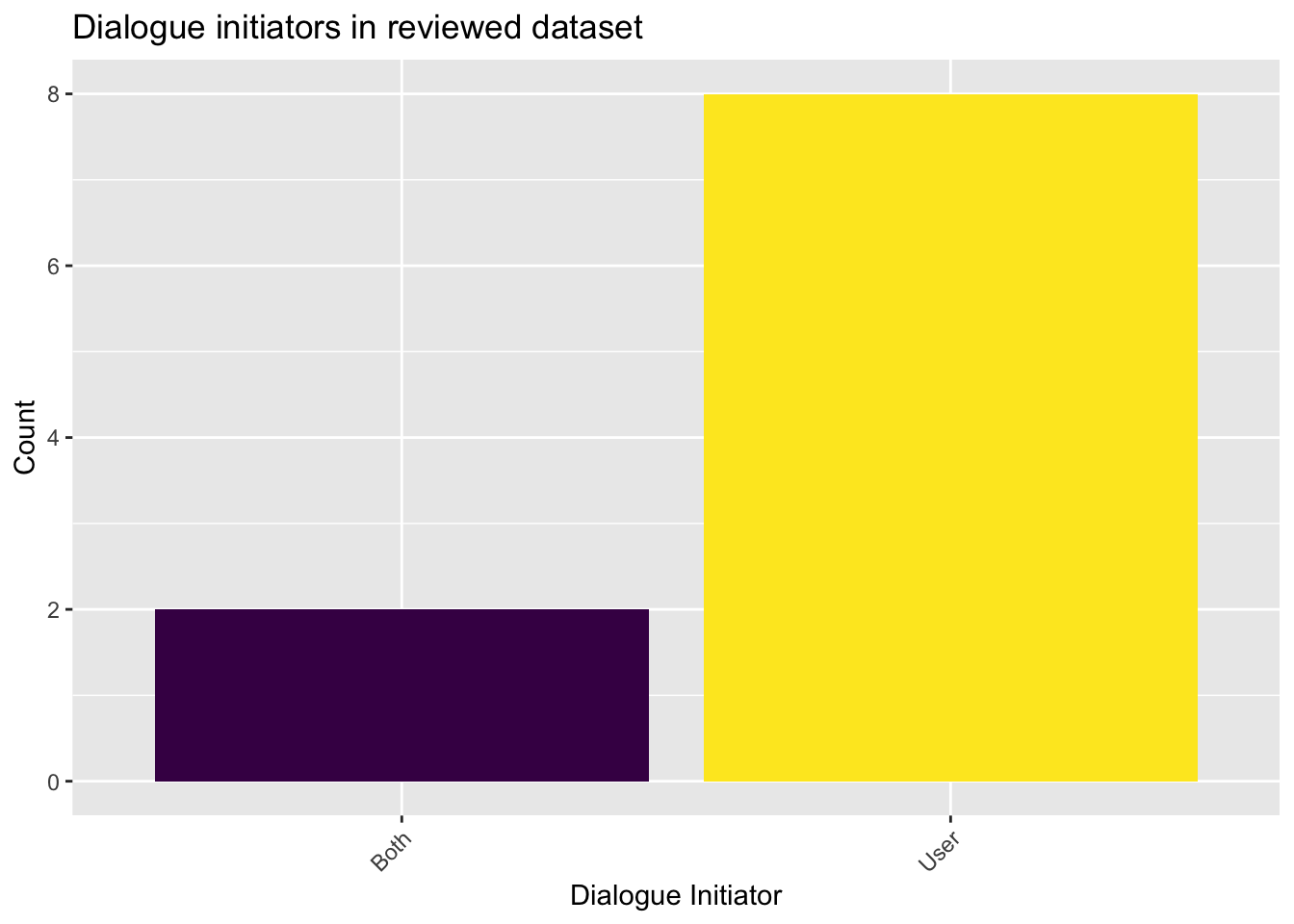

Supplement: Multimedia Appendix 1 [file ai-v5-e80123-s001.zip › supplementary_materials_large_language_models_pa_ct_scoping_review/05_data_analysis/052_quantitative_analysis/0523_data_analysis_figures/bar-dialogue-initiative-1.png]
